# Supplementary material for: Valuing conservation and natural wealth: The blue economy of manta ray watching in the Maldives
Source: PLoS One. 2026 Jun 1;21(6):e0326719. doi: 10.1371/journal.pone.0326719 (PMC13225372; doi:10.1371/journal.pone.0326719)
Supplement: S1 Table — Adapted from O’Malley et al. [3]. (DOCX) [file pone.0326719.s001.docx]

## S1 Table.

| **Term** | **Abbreviation** | **Definition** | **Source** |
| --- | --- | --- | --- |
| Geographical atoll |  | Atolls with distinct reef systems. These are grouped into administrative regions. | Ministry of Fisheries & Agriculture [73] |
| Administrative regions |  | Administrative regions (also known as atolls) are used to organise the country for governance. | Ministry of Fisheries & Agriculture [73] |
| Manta ray watching | MRW | Recreational activities of diving, snorkelling, and on-water observation with the intent of viewing manta rays in the wild, consistent with the criteria used in other studies. | O’Malley et al. [3] |
| Manta ray watching site |  | Manta ray sites are areas where manta ray sightings are known to be frequent seasonally, periodically, or year-round. Sites where manta rays were encountered opportunistically, or out of season, were not included as MRW sites because they do not fit the conservative approach of this study. | O’Malley et al. [3] |
| Manta ray watching guest | MRW guest | Are those who participated in a MRW trip, which may include individuals that participated in multiple trips. |  |
| Trip |  | A trip is a single dive or snorkel session with manta rays taken by guests on a vessel. For example, a two-tank dive counts as two trips. |  |
| Tour operator |  | Tour operators are dive and excursion activity centres (i.e., excursion centres offer recreational activities including snorkelling trips), as well as liveaboard vessels. Tour operators function on bases (i.e., resorts, liveaboard and community islands). |  |
| Tourism base | Base | Bases (i.e., resorts, liveaboards and community islands) are the actual venues in where tour operators operate their activity centres from. |  |
| Activity centre |  | In-water activities like diving and snorkelling are ran out of activity centres. |  |
| Excursion centre |  | These businesses offer recreational activities including snorkelling trips. |  |
| Inhabited community island | Community (or comm.) island | These are islands where the general population resides (i.e., towns, villages, fishing and farming communities with permanent human habitation). Some of these islands (especially ones where tourism is present) have dive and excursion activity centres. |  |
| Regular Resort Island | Resort | A < - (star) resort that operates under the concept “one-island-one-resort”, however does not meet the luxury standards and therefore has not been awarded a 5-star rating. |  |
| 5-star Luxury Resort Island | 5-star Resort (5* in figures) | A resort that operates under the concept “one-island-one-resort” and does meet the luxury standards and therefore has been awarded a 5-star rating. |  |
| Liveaboard |  | This is a vessel that has been designed for people to live aboard (multiple-day trips). It is generally used for recreational diving expeditions where divers onboard stay on the vessel for the duration of the cruise and use it as a diving support vessel. The guests onboard pay per person; thus, it is not a private charter. |  |
| Private liveaboard |  | Similar to a liveaboard, but the guests pay for the boat, not per person; thus, it is a private charter. A multi-day cruise on private liveaboards was generally priced between US$12,175–84,000. Because of these high prices, it was not possible to decipher the price per dive and thus, price per MRW dive. |  |
| Guesthouse |  | Guesthouses provide accommodation for tourists (domestic and international) and are generally at a lesser price than a resort. The vast majority do not function as tour operators and outsource snorkelling and diving activities to other businesses (e.g., dive and excursion centres); thus, were excluded |  |
