# Supplementary material for: Valuing conservation and natural wealth: The blue economy of manta ray watching in the Maldives
Source: PLoS One. 2026 Jun 1;21(6):e0326719. doi: 10.1371/journal.pone.0326719 (PMC13225372; doi:10.1371/journal.pone.0326719)
Supplement: S1 Appendix — Survey questions sent to active land-based operators (i.e., activity centres in resorts and on community islands) and one for boat-based operators (i.e., liveaboards). (DOCX) [file pone.0326719.s002.docx]

## S2 Table.

| **Details** | **Shorthand in equation** | **Collection date** | **Ministry of Tourism [49,50]** | **Maldives Bureau of Statistics [87]** | **Internet research** | **Tour operator surveys** | **Ministry of Tourism**  **[93]** | **Baa Atoll Biosphere Reserve [85]** | **Other sources*** |
| --- | --- | --- | --- | --- | --- | --- | --- | --- | --- |
| Tour operators in the Maldives |  | 2021-2022 | X |  | X | X |  |  |  |
| Tour operator revenue | TOR | 2022 |  |  | X | X |  |  |  |
| Activity price (US$) | AP | 2022-2023 |  |  | X | X |  |  |  |
| Number of guests per trip | NG | 2022 |  |  |  | X |  |  |  |
| Number of trips | NT | 2022 |  |  |  | X |  |  |  |
| Number of weeks of MRW season | NW | 2022-2023 |  |  | X | X |  |  | X |
| Number of opportunities for MRW | NO | 2022 |  |  |  | X |  |  |  |
| Manta ray watching sites |  | 2022 |  |  | X | X |  |  |  |
| Tourist expenses | TE | 2024 |  |  | X |  |  |  |  |
| Government TAX (US$) | TX | 2022 |  | X |  |  |  |  |  |
| Staff service charge (US$) | SSC | 2022 |  | X |  |  |  |  |  |
| Staff salary revenue (Maldivian and Foreign) (US$) | SSR_M or F_ | 2022 |  |  |  | X |  |  | X |
| Entry fees to MPA (Resident and Visitor) | EF_R or V_ | 2022 |  |  |  |  |  | X |  |
| Tour guide licence fee (US$) | TGL | 2022 |  |  |  |  |  | X |  |
| Professional partnerships fee (US$) | PP | 2022 |  |  |  |  |  | X |  |
| Videography permit fee (US$) | VP | 2022 |  |  |  |  |  | X |  |
| Gross Domestic Product (US$) | GDP | 2022-2023 | X | X |  |  | X |  |  |
| Manta ray (M. alfredi and M. birostris) population size | P | 2024 |  |  |  |  |  |  | X |
| Manta ray (M. alfredi and M. birostris) lifespan | LS | 2024 |  |  |  |  |  |  | X |

*Other sources include manta ray researchers, logbook data and published literature.
