# Supplementary material for: Valuing conservation and natural wealth: The blue economy of manta ray watching in the Maldives
Source: PLoS One. 2026 Jun 1;21(6):e0326719. doi: 10.1371/journal.pone.0326719 (PMC13225372; doi:10.1371/journal.pone.0326719)
Supplement: S2 Table — (DOCX) [file pone.0326719.s003.docx]

## S1 Appendix.

## Land-based and Boat-based Tour Operators Survey

*The Manta Trust – 2021*

Note*Only for the year 2021

### 1. Submitter details – your operation

**Name**

*Open*

**What gender do you identify as?**

*Category*

Female/Male/Prefer not to say

**Are you Maldivian National?**

*Category*

Y/N/Prefer not to say

**What is your age?**

*Category*

18-29 year / 30-39 years / 40 – 49 years / 50-59 years / 60-69 years / Prefer not to say

**How many years’ experience do you have in the diving/excursion industry in Maldives?**

*Numerical*

0-60 years

**What is the name of your operation?**

*Open*

**Which Atoll is your operation based in?**

*Category*

20 Atolls

**Where is your operation based?**

*Category*

Resort Island / Local Island

**Please select the option which best matches the type of operator you are (Please only answer questions for your operation. For example, if you are a third-party dive centre operating at a resort, only answer the questions about guests you serve at the dive centre)**.

*Category*

Guest house / Dive centre / Recreation, Excursions centre / Watersports / Sports centre

**Please select the option which best describes your PRIMARY job role in this operation in 2021:**

*Category*

Excursions, Watersports, Snorkel guide / Surf guide/Instructor / Divemaster / Dive Instructor / Marine Biologist / Assistant Manager / Manager

**Did your operation run manta ray focused trips in 2021? If so, which type of operator are you? (Please select the correct option to be directed to the questionnaire that suits your trip type)**

*Category – answer sends them to appropriate section of the survey*

No, we did not sell manta ray focused trips

Landbased operator selling manta ray focusing SNORKEL trips

Landbased operator selling manta ray focusing DIVE trips

Landbased operator selling manta ray focusing SNORKEL and DIVE trips

Liveaboard selling manta ray focused DIVES trips

### 2. a) Economics – Dive tours

#### Land-based tour operators only

**How many DIVERS were on an average MANTA ray TRIP in 2021?**

*Numerical*

0-40 pax and Unknown

**Please estimate how many DIVING TRIPS you made per WEEK on AVERAGE to MANTA ray sites?**

*Numerical*

0-14 trips and Unknown

**What was the cost per GUEST per manta ray DIVE in USD$?**

*Open*

Short answer

**In 2021, how many WEEKS long was the season when your operator could offer DIVING trips to MANTA ray specific sites?**

*Numerical*

0-52 weeks and Unknown

**Did your operator offer any manta ray distinctive specialty diver course?**

*Category*

Y/N

**If yes, how many manta ray distinctive specialty diver courses were conducted in 2021 and what was the price per GUEST in USD$?**

*Open*

Short answer

**Did your operator offer a manta ray presentation for guests to attend?**

*Category*

Y/N

**If yes, what was the cost per guest in USD$? (please write '0' if it was free)**

*Open*

Short answer

**Please write the total estimated number of guests that attended the presentation in 2021:**

*Open*

Short answer

### 2. b) Economics – Snorkel tours

#### Land-based tour operators only

**How many SNORKELLERS were on an average MANTA ray TRIP in 2021?**

*Numerical*

0-40 pax and Unknown

**Please estimate how many SNORKELLING TRIPS you made per WEEK on AVERAGE to MANTA ray sites?**

*Numerical*

0-14 trips and Unknown

**What was the cost per GUEST per manta ray SNORKEL in USD$?**

*Open*

Short answer

**In 2021, how many WEEKS long was the season when your operator could offer SNORKEL excursions to MANTA ray specific sites?**

*Numerical*

0-52 weeks and Unknown

**Did your operator offer a manta ray presentation for guests to attend?**

Y/N

**If yes, what was the cost per guest in USD$? (please write '0' if it was free)**

*Open*

Short answer

**Please write the total estimated number of guests that attended the presentation in 2021:**

*Open*

Short answer

### 2. c) Economics – liveaboards

#### Boat-based operators only

**What was the most common trip length on your liveaboard in 2021?**

*Category*

Mostly 7 NIGHT trips

Mostly 10 NIGHT trips

Mostly 14 NIGHT trips

Unknown

**What was the average number of TRIPS run per MONTH on your liveaboard in 2021?**

*Numerical*

0-10 trips and Unknown

**What was the average cost per GUEST per NIGHT on a TRIP on your liveaboard in 2021 in USD$? (Please write "UNKNOWN" if the cost is unknown)**

*Open*

Short answer

**How many GUESTS were on your liveaboard on an average TRIP in 2021?**

*Numerical*

0-30 pax and Unknown

**How many WEEKS were NO trips conducted on your liveaboard in 2021? (e.g. no guests, time spent on maintenance, boat was somewhere other than in the Maldives)**

*Numerical*

0-52 weeks and Unknown

**On an average TRIP in 2021, how many DIVES would your liveaboard do?**

*Numerical*

0-50 dives and Unknown

**On an average TRIP in 2021, how many MANTA ray focused DIVES would your liveaboard do?**

*Numerical*

0-40 dives and Unknown

**On an average TRIP in 2021, how regularly did you see MANTA rays**?

*Category*

<25% of trips

25-50% of trips

50-75% of trips

75-90% of trips

>90% of trips

Unknown

**Please estimate on how many TRIPS in 2021 your liveaboard did NOT visit MANTA ray sites?**

*Numerical*

0-60 trips and Unknown

**Did your liveaboard offer any SNORKEL excursions to MANTA ray specific sites in 2021?**

*Category*

Yes/No/Unknown

**If yes, on average how many SNORKEL excursions were offered during a trip, and to which manta ray sites (please LIST sites in order of most visited and the atolls where they are located)?**

*Open*

Short answer

**Please select all the atolls that your liveaboard visited to see manta rays in the NORTHEAST Monsoon (IRUVAI)?**

*Category*

Northern Atolls (Haa Alifu, Haa Dhaalu, Noonu, Shaviyani)

Raa Atoll (Inc. Kalhifushi)

Baa Atoll (Inc. Goidhu & Fasdhūetherē)

Lhaviyani Atoll

North & South Malé Atolls (Inc. Gaafaru & Kaashidhoo)

Ari Atoll (Inc. Thoddu & Rasdhu)

Vaavu Atoll (Inc. Vattaru)

Faafu and Dhaalu

Meemu Atoll

Thaa Atoll

Laamu Atoll

Gaafu Atoll

Fuvahmulah Atoll

Addu Atoll

Unknown

**Please select all the atolls that your liveaboard visited to see manta rays in the SOUTHWEST Monsoon (HULHANGU)?**

*Category*

Northern Atolls (Haa Alifu, Haa Dhaalu, Noonu, Shaviyani)

Raa Atoll (Inc. Kalhifushi)

Baa Atoll (Inc. Goidhu & Fasdhūetherē)

Lhaviyani Atoll

North & South Malé Atolls (Inc. Gaafaru & Kaashidhoo)

Ari Atoll (Inc. Thoddu & Rasdhu)

Vaavu Atoll (Inc. Vattaru)

Faafu and Dhaalu

Meemu Atoll

Thaa Atoll

Laamu Atoll

Gaafu Atoll

Fuvahmulah Atoll

Addu Atoll

Unknown

**Did your operator offer any manta ray distinctive specialty diver course?**

*Category*

Y/N

**If yes, how many manta ray distinctive specialty diver courses were conducted in 2021 and what was the price per GUEST in USD$?**

*Open*

Short answer

**Did your operator offer a manta ray presentation for guests to attend?**

*Category*

Y/N

**If yes, what was the cost per guest in USD$? (please write '0' if it was free)**

*Open*

Short answer

**Please write the total estimated number of guests that attended the presentation in 2021:**

*Open*

Short answer

**If your liveaboard outsourced manta ray DIVE or SNORKEL trips to another operator in 2021, please write the operators name below:**

*Open*

Short answer

### 3. Sightings

**Which species of manta ray do you see most frequently on your snorkel tours?**

*Category*

Reef manta ray

Oceanic (giant) manta ray

Unknown

We don’t see manta rays

**Please LIST the names (and the atolls where they are located) of the FIVE manta ray SNORKEL sites your operator visited MOST frequently in 2021 (in order of most visited first).**

*Open*

Short answer

**Please LIST the estimated average number of MANTA RAYS present during a single SNORKEL in 2021 at EACH of the five sites listed in the previous question (in the same order as previously).**

*Open*

Short answer

**Please LIST the estimated average number of TOURIST BOATS present during a single SNORKEL in 2021 at EACH of the five sites listed in the earlier question (in the same order as previously).**

*Open*

Short answer

**Please LIST the estimated average number of PAYING TOURISTS (including those from other operators) present during a single SNORKEL in 2021 at EACH of the five sites listed in the earlier question (in the same order as previously).**

*Open*

Short answer

### 4. Socio-economic and intrinsic value

**If your operator outsourced manta ray DIVE or SNORKEL trips to another operator in 2021 please write the operators name below:**

*Open*

Short answer

**Where do you think manta rays ranked among sea life divers/snorkellers most wanted to see?**

*Category*

Number 1 animal divers wanted to see / In the top 2 / In the top 3 / In the top 4 / In the top 5 / NOT in the top 5 / Unknown

**How do manta ray's feature at your operation? (Please select all that apply and tell us about extras using the 'other' option)**

*Category*

Manta ray feature in our logo

Manta ray photos/videos on the website/brochures

Marketing mentions famous manta ray diving/snorkelling sites nearby our operation

Manta ray shaped structure or building

Manta ray art (decorations, wall art, sand art)

Manta ray merchandise (e.g. book, dive/snorkel gear, clothing, jewellery)

Manta rays do not feature in our operation

Unknown

**Do you consider manta rays to be important to your operator's business?**

*Category*

Y/N

**Please tell us why/why not you consider manta rays to be important to your operation's business?**

*Open*

Short answer

**Do you think manta rays are important to local communities in the Maldives?**

*Category*

Y/N/Unknown

**Please tell us why/why not you consider manta rays to be important to local communities in the Maldives.**

*Open*

Short answer

**Do you think that your manta ray trips helped to educate guests about manta rays and conservation? How?**

*Open*

Short answer

**How many staff in your department regularly worked on manta ray focused trips in 2021? This includes snorkel/dive guides, boat crew/captains, office staff etc.**

*Numerical*

0-40 staff and Unknown

**Please estimate how many MALDIVIAN staff in your department regularly worked on manta ray focused trips in 2021? This includes snorkel/dive guides, boat crew/captains, office staff etc.**

*Numerical*

0-40 staff and Unknown

**Please estimate how COVID-19 impacted your operation in 2021?**

*Category*

Not at all / 25% reduction in sales / 50% reduction in sales / 75% reduction in sales / Not currently operational due to COVID-19 / Unknown

**When did your operator re-open after COVID-19 closures? (If your operator never closed, please write this below)**

*Open*

Short answer
