# Supplementary material for: Valuing conservation and natural wealth: The blue economy of manta ray watching in the Maldives
Source: PLoS One. 2026 Jun 1;21(6):e0326719. doi: 10.1371/journal.pone.0326719 (PMC13225372; doi:10.1371/journal.pone.0326719)
Supplement: Attachment — Submitted filename: Review MI.docx [file pone.0326719.s004.docx]

Valuing conservation and natural wealth: The blue economy of manta ray watching in the Maldives

--Manuscript Draft Review

123: Could be a separate paragraph about threats and challenges.

97-100 and 131-132:

Please explain the magnitude of shark fishing in the Maldives. Is it mainly bycatch or targeted? And when did it start?

What species of shark were extracted? If it is a specialised shark fishery, the fishers tend to extract specific species and sizes to maximise the catch (fins are usually the main body parts in targeted shark fisheries) such as adult thresher, silky shark, hammerhead, etc and specialised fishers usually using longlines that mostly operates in open water instead of the reefs /coast where marine tourism (such as diving) took place. If the sharks at dive sites, such as reef and tiger sharks (which targeted shark fishers do not prioritise), are declining, it is not necessarily because of specialised shark fisheries. See Mustika et al. (2018) <https://doi.org/10.3389/fmars.2020.00261> for more information. Also, declining visit numbers in a country’s tourism might be a combination of several factors such as political instability, rising commodity prices, and natural disasters.

136-137*:* 3,500 individuals are from mark-recapture analysis; what about the 6,200 individuals? Please elaborate to avoid confusion.

298-304: Liveaboard trips’ diving numbers are hard to quantify, since the number of dives at manta sites is determined by customer requests, and some divers may dive more than others. Are there any other non-manta-ray diving sites with significant visitation?

371: For the estimated lifespan, I suppose authors use the whole lifespan, as used in many references.

I think it would be interesting to consider what age/size manta rays are occupied by or seen at the diving sites as a marine tourism highlight? Are the younger, smaller manta rays seen often at the main dive sites? Do they have separate aggregation sites before they reach maturity (about 8 years)?

See Setyawan et. al. 2022a <https://doi.org/10.3389/fmars.2022.815094>

385-386: While *M. alfredi* move locally between seasons, and *M. birostris* MRW in Fuvahmulah (Table 1) are active only in the NE monsoon, do we have any information (for example, from tagging studies) on where they are for the rest of the year?

387-390: With only eight responses from 13% of completed surveys among liveaboards, I will be cautious in estimating revenue (4%) and state that liveaboards mostly visit Fuvahmulah. If there is more information/reference regarding this, please add it to the MS.

419: Does the number of boats per trip mean the number of boats sighted at a time in a particular site? Please elaborate.

420: How to define a trip? For manta rays sighted per trip, is it more accurate if it’s manta rays per dive? And do you have information on how many dives per trip? Please state in the MS.

421: “Encounters with *M. birostris* were only promoted in Fuvahmulah”, already mentioned in the previous section. Consider removing or combining it with the previous sentence.

523-525: Interesting point. Do you have more specific information about manta rays’ cultural value within the community? Any cultural products, such as myths, legends, and Folklore, as found in many indigenous societies around the world? This value, if communicated well, can strengthen a sense of identity and support conservation.

537: You can also explain the response on “how manta rays ranked among marine life visitors wanted to see” in a sentence instead of in Figure 5.

597: You can add more examples from regions where manta rays or other elasmobranch species are valued as a marine tourism spectacle but also facing threats of unsustainable fishing (e.g., Indonesia, Philippines). See O’Malley 2013 <https://journals.plos.org/plosone/article/file?id=10.1371/journal.pone.0065051&type=printable>.

Also, be mindful of stating that fisheries mortality contributes to the decline in elasmobranch species at dive sites, since many specialised elasmobranch fisheries target specific species and sizes, and operate offshore rather than in tourism spots, as I mentioned in the previous section.

When comparing MRW management across countries, it is helpful to include additional context on each country’s size and management to provide readers with more information. For example, the Raja Ampat region in Indonesia alone is a 40,000 km² Regency, with several manta ray dive sites and 51 manta-sighting sites, managed as a network of five MPAs by the local MPA Network Management Unit (BLUD), which might differ from the Maldives’ centralised MPA management.

See Setyawan 2022b <https://doi.org/10.3389/fmars.2022.1014791>

662: You might want to start with the negative impacts on manta rays. Or start the paragraph with “When tourism is unmanaged or is beyond the carrying capacity of an area, the industry is unsustainable and requires further regulation and enforcement”, and give examples of manta rays and cetaceans.

724: Similar to lines 523-525, please elaborate more about “natural goodness”, which might be very interesting for the readers.

**Why the paper is good**

This MS is well written, and the authors successfully highlight its value. First of all, as an updated study of the economic value of Maldives MRW after more than a decade is very important for future management. This study also highlights the discrepancy in staff salary revenue between Maldivian and expatriate staff, as well as the need for more stable funding that supports in-country conservation from tourism. Further, this study is also very important for backing arguments against unsustainable fishing practices (such as the recent plan to legalise shark fishing in the Maldives in 2024) and any future threats to biodiversity. Minor improvements are needed, including elaboration on fisheries and cultural significance.
