# Supplementary material for: Valuing conservation and natural wealth: The blue economy of manta ray watching in the Maldives
Source: PLoS One. 2026 Jun 1;21(6):e0326719. doi: 10.1371/journal.pone.0326719 (PMC13225372; doi:10.1371/journal.pone.0326719)
Supplement: Attachment — Submitted filename: Response to Reviewers.docx [file pone.0326719.s006.docx]

# PLOS ONE – major revisions

**Contents of letter “Response to Reviewers”**

Table of Contents

[PLOS ONE – major revisions	1](#_Toc219636412)

[Letter from Editor	1](#_Toc219636413)

[Comments to the Author	7](#_Toc219636414)

[Reviewer #1	8](#_Toc219636415)

[Reviewer #2	21](#_Toc219636416)

## Letter from Editor

| **Date:** | Dec 05 2025 10:06PM |
| --- | --- |
| **To:** | "Hannah Maree Moloney" hannah.moloney@mantatrust.org;hannah.moloney@research.usc.edu.au |
| **From:** | "PLOS ONE" plosone@plos.org |
| **Subject:** | PLOS ONE Decision: Revision required [PONE-D-25-29750] |
| **Attachment(s):** | [Review MI.docx](https://www.editorialmanager.com/pone/download.aspx?guid=%7B24836857-afcc-4bdc-b865-a12280bd9932%7D&scheme=13&id=597121) |

PONE-D-25-29750

Valuing conservation and natural wealth: The blue economy of manta ray watching in the Maldives

PLOS One

Dear Dr. Moloney,

Thank you for submitting your manuscript to PLOS ONE. After careful consideration, we feel that it has merit but does not fully meet PLOS ONE’s publication criteria as it currently stands. Therefore, we invite you to submit a revised version of the manuscript that addresses the points raised during the review process.

Please submit your revised manuscript by Jan 19 2026 11:59PM. If you will need more time than this to complete your revisions, please reply to this message or contact the journal office at [plosone@plos.org](mailto:plosone@plos.org). When you're ready to submit your revision, log on to https://www.editorialmanager.com/pone/ and select the 'Submissions Needing Revision' folder to locate your manuscript file.

Please include the following items when submitting your revised manuscript:

- A rebuttal letter that responds to each point raised by the academic editor and reviewer(s). You should upload this letter as a separate file labeled 'Response to Reviewers'.
- A marked-up copy of your manuscript that highlights changes made to the original version. You should upload this as a separate file labeled 'Revised Manuscript with Track Changes'.
- An unmarked version of your revised paper without tracked changes. You should upload this as a separate file labeled 'Manuscript'.

If you would like to make changes to your financial disclosure, please include your updated statement in your cover letter. Guidelines for resubmitting your figure files are available below the reviewer comments at the end of this letter.

If applicable, we recommend that you deposit your laboratory protocols in protocols.io to enhance the reproducibility of your results. Protocols.io assigns your protocol its own identifier (DOI) so that it can be cited independently in the future. For instructions see: <https://journals.plos.org/plosone/s/submission-guidelines#loc-laboratory-protocols>. Additionally, PLOS ONE offers an option for publishing peer-reviewed Lab Protocol articles, which describe protocols hosted on protocols.io. Read more information on sharing protocols at <https://plos.org/protocols?utm_medium=editorial-email&utm_source=authorletters&utm_campaign=protocols>.

We look forward to receiving your revised manuscript.

Kind regards,

Joel Harrison Gayford
Academic Editor
PLOS One

**Response:** Thank you for giving us the opportunity to resubmit our manuscript. We have addressed the feedback and provided point by point detailed replies below and in the “Response to Reviewers” letter.

**Journal Requirements:**

When submitting your revision, we need you to address these additional requirements.

1.Please ensure that your manuscript meets PLOS ONE's style requirements, including those for file naming. The PLOS ONE style templates can be found at

https://journals.plos.org/plosone/s/file?id=wjVg/PLOSOne_formatting_sample_main_body.pdf and

<https://journals.plos.org/plosone/s/file?id=ba62/PLOSOne_formatting_sample_title_authors_affiliations.pdf>

**Response:** Changed. The manuscript now complies with PLOS ONE’s style template.

2. When completing the data availability statement of the submission form, you indicated that you will make your data available on acceptance. We strongly recommend all authors decide on a data sharing plan before acceptance, as the process can be lengthy and hold up publication timelines. Please note that, though access restrictions are acceptable now, your entire data will need to be made freely accessible if your manuscript is accepted for publication. This policy applies to all data except where public deposition would breach compliance with the protocol approved by your research ethics board. If you are unable to adhere to our open data policy, please kindly revise your statement to explain your reasoning and we will seek the editor's input on an exemption. Please be assured that, once you have provided your new statement, the assessment of your exemption will not hold up the peer review process.

**Response:** Changed. Data is now available through Zenodo using DOI [10.5281/zenodo.18252206](https://doi.org/10.5281/zenodo.18252206)

3. Your ethics statement should only appear in the Methods section of your manuscript. If your ethics statement is written in any section besides the Methods, please move it to the Methods section and delete it from any other section. Please ensure that your ethics statement is included in your manuscript, as the ethics statement entered into the online submission form will not be published alongside your manuscript.

**Response:** Changed. We have removed the ethics statement from the *Acknowledgment* section and moved it to the *Methods* (under Study Design, Tour operator surveys):

“The research was carried out under the *Environmental Protection Agency* Protected Species Research Permit (*EPA/2021/PSR-M09*) and in accordance with the University of the Sunshine Coast Human Ethics exemption (*E24002*).”

4. We note that Figure 1 in your submission contain map images which may be copyrighted. All PLOS content is published under the Creative Commons Attribution License (CC BY 4.0), which means that the manuscript, images, and Supporting Information files will be freely available online, and any third party is permitted to access, download, copy, distribute, and use these materials in any way, even commercially, with proper attribution. For these reasons, we cannot publish previously copyrighted maps or satellite images created using proprietary data, such as Google software (Google Maps, Street View, and Earth). For more information, see our copyright guidelines: http://journals.plos.org/plosone/s/licenses-and-copyright.

We require you to either (1) present written permission from the copyright holder to publish these figures specifically under the CC BY 4.0 license, or (2) remove the figures from your submission:

1. You may seek permission from the original copyright holder of Figure 1 to publish the content specifically under the CC BY 4.0 license.

We recommend that you contact the original copyright holder with the Content Permission Form (http://journals.plos.org/plosone/s/file?id=7c09/content-permission-form.pdf) and the following text:

“I request permission for the open-access journal PLOS ONE to publish XXX under the Creative Commons Attribution License (CCAL) CC BY 4.0 (http://creativecommons.org/licenses/by/4.0/). Please be aware that this license allows unrestricted use and distribution, even commercially, by third parties. Please reply and provide explicit written permission to publish XXX under a CC BY license and complete the attached form.”

Please upload the completed Content Permission Form or other proof of granted permissions as an "Other" file with your submission.

In the figure caption of the copyrighted figure, please include the following text: “Reprinted from [ref] under a CC BY license, with permission from [name of publisher], original copyright [original copyright year].”

2. If you are unable to obtain permission from the original copyright holder to publish these figures under the CC BY 4.0 license or if the copyright holder’s requirements are incompatible with the CC BY 4.0 license, please either i) remove the figure or ii) supply a replacement figure that complies with the CC BY 4.0 license. Please check copyright information on all replacement figures and update the figure caption with source information. If applicable, please specify in the figure caption text when a figure is similar but not identical to the original image and is therefore for illustrative purposes only.

The following resources for replacing copyrighted map figures may be helpful:

USGS National Map Viewer (public domain): http://viewer.nationalmap.gov/viewer/

The Gateway to Astronaut Photography of Earth (public domain): http://eol.jsc.nasa.gov/sseop/clickmap/

Maps at the CIA (public domain): https://www.cia.gov/library/publications/the-world-factbook/index.html and https://www.cia.gov/library/publications/cia-maps-publications/index.html

NASA Earth Observatory (public domain): http://earthobservatory.nasa.gov/

Landsat: http://landsat.visibleearth.nasa.gov/

USGS EROS (Earth Resources Observatory and Science (EROS) Center) (public domain): http://eros.usgs.gov/#

Natural Earth (public domain): <http://www.naturalearthdata.com/>

**Response:** Changed. Thank you for the suggestions. We were able to locate the reference for the spatial data we have used through the Ocean Plus website <https://habitats.oceanplus.org>. We have also contacted Ocean Plus via email to request additional permissions using PLOS ONE’s “Content-permission-form”, however we are yet to receive a response.

We have updated the figure caption to contain all reference material:

**“Fig. 1. Geographical distribution of the 10 primary manta ray watching sites (shown by red circles) in the Republic of Maldives’. The 26 geographical atolls of the Maldives’ archipelago are shown by the red box in panel a. The capital, Malé City is identified by the yellow triangle and the nation’s 20 administrative divisions are highlighted in blue in panel b.** Although Malé City is an administrative division, for this study we have grouped it with Kaafu, thus, only 20 divisions are shown. The manta ray watching sites correspond with those outlined in Table 2. This map was created in R Studio using reef features from Millennium Coral Reef Mapping Project (MCRMP; <https://habitats.oceanplus.org>); MCRMP validated maps provided by the Institute for Marine Remote Sensing, University of South Florida (IMaRS/USF) and Institut de Recherche pour le Développement (IRD, Centre de Nouméa), with support from NASA. IRD does not endorse these products [75].”

**REFERENCE:**

UUNEP-WCMC. Ocean+ Habitats [On-line]. 2020. doi:https://doi.org/10.34892/fpe3-ar97

5. We note that there is identifying data in the Supporting Information file < S1 Appendix.docx>. Due to the inclusion of these potentially identifying data, we have removed this file from your file inventory. Prior to sharing human research participant data, authors should consult with an ethics committee to ensure data are shared in accordance with participant consent and all applicable local laws.

Data sharing should never compromise participant privacy. It is therefore not appropriate to publicly share personally identifiable data on human research participants. The following are examples of data that should not be shared:

-Name, initials, physical address

-Ages more specific than whole numbers

-Internet protocol (IP) address

-Specific dates (birth dates, death dates, examination dates, etc.)

-Contact information such as phone number or email address

-Location data

-ID numbers that seem specific (long numbers, include initials, titled “Hospital ID”) rather than random (small numbers in numerical order)

Data that are not directly identifying may also be inappropriate to share, as in combination they can become identifying. For example, data collected from a small group of participants, vulnerable populations, or private groups should not be shared if they involve indirect identifiers (such as sex, ethnicity, location, etc.) that may risk the identification of study participants.

Additional guidance on preparing raw data for publication can be found in our Data Policy (https://journals.plos.org/plosone/s/data-availability#loc-human-research-participant-data-and-other-sensitive-data) and in the following article: http://www.bmj.com/content/340/bmj.c181.long.

Please remove or anonymize all personal information (<specific identifying information in file to be removed>), ensure that the data shared are in accordance with participant consent, and re-upload a fully anonymized data set. Please note that spreadsheet columns with personal information must be removed and not hidden as all hidden columns will appear in the published file.

**Response:** Changed. We have removed all personal data from <S1 Appendix.docx >.

6. If the reviewer comments include a recommendation to cite specific previously published works, please review and evaluate these publications to determine whether they are relevant and should be cited. There is no requirement to cite these works unless the editor has indicated otherwise. 

**Response:** Thank you for clarifying.

Additional Editor Comments:

You will see that both reviewers generally have a favourable view of the manuscript, but that some changes are required. Please take care to address all of the points raised by the reviewers when making your revision. 

**Response:** Thank you also for your feedback and suggestions. We have taken the time to carefully address each point made by both reviewers and the editor.

Comments to the Author

**1. Is the manuscript technically sound, and do the data support the conclusions?**

The manuscript must describe a technically sound piece of scientific research with data that supports the conclusions. Experiments must have been conducted rigorously, with appropriate controls, replication, and sample sizes. The conclusions must be drawn appropriately based on the data presented.

Reviewer #1: No

Reviewer #2: Yes

**2. Has the statistical analysis been performed appropriately and rigorously?**

Reviewer #1: No

Reviewer #2: Yes

**3. Have the authors made all data underlying the findings in their manuscript fully available?**

The [PLOS Data policy](http://www.plosone.org/static/policies.action#sharing) requires authors to make all data underlying the findings described in their manuscript fully available without restriction, with rare exception (please refer to the Data Availability Statement in the manuscript PDF file). The data should be provided as part of the manuscript or its supporting information, or deposited to a public repository. For example, in addition to summary statistics, the data points behind means, medians and variance measures should be available. If there are restrictions on publicly sharing data—e.g. participant privacy or use of data from a third party—those must be specified.

Reviewer #1: No

Reviewer #2: Yes

**Response:** Changed. Data is now available through Zenodo using DOI [10.5281/zenodo.18252206](https://doi.org/10.5281/zenodo.18252206)

**4. Is the manuscript presented in an intelligible fashion and written in standard English?**

PLOS ONE does not copyedit accepted manuscripts, so the language in submitted articles must be clear, correct, and unambiguous. Any typographical or grammatical errors should be corrected at revision, so please note any specific errors here.

Reviewer #1: Yes

Reviewer #2: Yes

## Reviewer #1

**5. Review Comments to the Author

Please use the space provided to explain your answers to the questions above. You may also include additional comments for the author, including concerns about dual publication, research ethics, or publication ethics. (Please upload your review as an attachment if it exceeds 20,000 characters)**

**Review of PONE-D-25-29750**

This article is an important contribution to the literature on the economic value of nature-based tourism. It serves as an important piece of information to highlight often hidden benefits of biodiversity conservation for local economies. There are significant shortcomings related to the methods used that need to be addressed before considering publication.

**Response:** Thank you for your positive feedback and helpful revisions particularly regarding the methods.

**1. Is the manuscript technically sound, and do the data support the conclusions?**
Due to methodological issues, the manuscript is not technically sound and does not support the conclusion in its current state. The manuscript requires major revisions in the methods to be considered for publication.

**Response:** Changed. We have addressed all points made during the revision process, particularly ones related to the *Methods* where changes were suggested.

**2. Has the statistical analysis been performed rigorously and appropriately?**
• The term “total economic value” or total economic benefit” is not appropriate. Check Randall 1987. You are only looking at direct use of the use value category.

**Response:** Changed. We have removed “total economic value” throughout the manuscript and replaced it with “direct economic benefit”. Thank you for your suggestion. Although we could not access the entirety of the [book](https://books.google.com.au/books?hl=en&lr=&id=XFaX0xhyLX4C&oi=fnd&pg=PA3&dq=The+total+value+dilemma:+toward+the+measurement+of+total+economic+value.&ots=8rIP_vDZAE&sig=6Q9D0fvxch2J82tmSQwS2tqb4lA#v=onepage&q=The%20total%20value%20dilemma%3A%20toward%20the%20measurement%20of%20total%20economic%20value.&f=false) suggested (Randall 1987), we agree that the term “total economic value” is not appropriate in the circumstance of this manuscript. We have reviewed another similar study and have used Zimmerhackel et al. (2019) as a basis for the economic language in our study. We have changed the language used in the *Methods* (equation descriptions and a caveat), *Results*, *Discussion* and *Abstract*.

*Methods:*

“The direct economic benefit from MRW tourism (*DEB_MRW_*_,_ US$*; Equation 1*) in the Maldives in 2021 was estimated as the sum of: i) the tour operator revenue (*TOR*) from manta-ray-specific snorkelling (*TOR_S_*, US$), diving (*TOR_D_*, US$), and diving from a liveaboard (*TOR_LAB_*, US$) activities; ii) the revenue from tourist expenses (*TE*, US$) including accommodation, food and beverages; iii) the revenue for government TAX (*TX*, US$) and staff service charge (*SSC*, US$) (proportions of *TOR+TE*); iv) staff salary revenue (*SSR*, US$) for those who work directly with MRW tourism; and v) the Hanifaru MPA revenue (*MPAR*, US$). Currency values throughout this study are presented as US$.”

***“Equation 1.*** *DEB_MRW_* *= TOR_S_ + TOR_D_ + TOR_LAB_ + TE + TX + SSC + SSR + MPAR”*

“The economic indicators in this study reflect direct financial flows providing a measure of the industry’s scale comparable to GDP. However, these equations do not represent the total economic value of MRW tourism, as they exclude consumer surplus, use and non-use values [86] and producer surplus (profits), and only represent the revenue generated [52].”

**REFERENCES**

Randall, A. 1987. The total value dilemma: toward the measurement of total economic value. Pages 3-13 in G. L. Peterson and C. F. Sorg, eds. U.S. For. Serv. Gen. Tech. Rep. RM-148.

<https://books.google.com.au/books?hl=en&lr=&id=XFaX0xhyLX4C&oi=fnd&pg=PA3&dq=The+total+value+dilemma:+toward+the+measurement+of+total+economic+value.&ots=8rIP_vDZAE&sig=6Q9D0fvxch2J82tmSQwS2tqb4lA#v=onepage&q=The%20total%20value%20dilemma%3A%20toward%20the%20measurement%20of%20total%20economic%20value.&f=false>

Zimmerhackel JS, Kragt ME, Rogers AA, Ali K, Meekan MG. Evidence of increased economic benefits from shark-diving tourism in the Maldives. Mar Policy. 2019;100: 21–26. doi:10.1016/j.marpol.2018.11.004

• Operator surveys: some questions are worded as if the questions were targeting the visitors, not the operators. “What was the cost per guest dive …?” is vague. Since it’s an operator survey, I assume you asked about operating costs, what the dives cost the operator, not cost the diver/customer. If so, why did you not specify subcategories to then more precisely model these expenditures in the wider economy? In any case, revenue = dives * price, not dives * cost.

**Response:** Thank you for your suggestion. To calculate the revenue, we have used revenue = dives * price. Despite the survey question using the term “cost” per dive, we can be sure that tour operators provided the “price” per guest as the explanation text that was provided in the email sent out alongside the survey further clarified the purpose of the survey. Additionally, the introduction text of the survey informed participants that we were seeking the economic value of manta ray tourism, not the cost to run manta ray trips or the profit made from trips. The survey participants were commonly dive instructors and expedition leaders who may not have the information on the operating cost per dive and are generally more focused on incoming revenue than overall profit. We additionally validated all survey answers using data mining.

• Given this issue, how sure are you that operators actually responded with their operating costs and not price charged, or vice versa? I think what you really were after was “price”, not “cost”, in order to calculate revenue. E.g. L239: In this sentence it looks like you used the term “cost” as meaning the price the operator charged for a package/experience/dive, etc.

**Response:** Changed. Yes we were seeking the “price” per activity not the “cost”. We can confirm that survey participants shared the “price” with us as it was clear in the written instructions that we were seeking the overall revenue per operator. The price was additionally validated using online data mining. We have changed the wording in all circumstances from “cost” to “price” in the manuscript when referring to the overall price of an activity, accommodation or liveaboard in *Methods* and *Results* (Including Table 6 and Supporting Information: S1 Table).

• For non-respondents, how did you determine the number of dives these non-responding operators had in the study year? On their websites, did you look for their capacity, e.g. number of vessels or dive guides, etc. to give you a better sense than just taking the average of whatever grouping the operator fell into? How did you verify your assumptions?

**Response:** Updated. We have included an explicit caveat in the *Discussion:*

“For non-responding operators, missing data were estimated via online data mining; where specific operation metrics were unavailable (e.g., number of weekly MRW dives or snorkels), we applied the mean values derived from responding operators of a similar category. While the level of uncertainty introduced by this extrapolation is unknown, it provided a necessary pathway for national-scale estimation.”

• L272 – L277: What if the tour operator didn’t update the website to reflect current prices? How did you deal with outliers? Did you employ any statistical approach to identify them? Citation missing.

**Response:** We took a conservative approach to not overinflate the value of manta ray tourism. Thus, if the website was not updated, it would have likely underestimated the price of the snorkelling and diving trips when accounting for inflation. This is now mentioned in the caveats section of the *Discussion*. In terms of outliers, especially from survey responses, we removed any obvious outliers (*n*=2) during the data visualisation stage.

*Methods:*

“To verify results from survey responses (i.e., activity price and season length), additional data mining was undertaken online for all participating tour operators. Surveys with major discrepancies between operator survey responses and internet data were excluded. For example, if the survey reported an activity price >20% different to that of the same company stated online, the value from the survey was disregarded. Obvious outliers in survey responses were excluded from this study in the initial data cleaning process (*n*=2).”

*Discussion:*

“Furthermore, in instances where tour operators had not recently updated their websites, the collected data likely underestimated the price of snorkelling and diving trips relative to current inflation. Consequently, we have maintained a conservative approach throughout the analysis to ensure the revenue generated by manta ray tourism is not overinflated.”

• With many experiences sold as packages, it’s unclear how you divided packages offered on websites into the categories you outlined in Equation 1.

**Response:** We have increased clarity on how this was achieved by clarifying equation 2b’s description.

Equation 1 includes the products from Equation 2a (snorkelling and diving MRW from a land-based tour operator) and 2b (MRW diving from a liveaboard). We didn’t use price packages in equation 2a, but the overall price of the liveaboard package was used in equation 2b (below).

*Methods:*

*“TOR_LAB_* (US$) was calculated as the product of the value of the activity price (*AP*, US$), the number of opportunities to snorkel and dive with manta rays (*NO*, unitless), the number of guests per trip (*NG*, unitless), and the number of trips per year in 2021 (*NT*, year^-1^; *Equation 2b*). As *TOR_LAB_* already accounted for tourist expenses (*TE*) as they are sold as a package, we estimated that only half of the price per trip (50%) was used to cover diving expenses and thus was used to calculate the activity price (*AP*) using the overall number of dives offered per liveaboard trip. We then used the activity price (*AP*) and the number of opportunities (*NO*) to snorkel and dive with manta rays to calculate the price per MRW dive. Where values for the activity price (*AP*), the number of guests (*NG*), the number of trips (*NT*), and the number of opportunities (*NO*) were missing from tour operators (e.g., non-responding operators), a mean value was assigned.”

***“Equation 2b.*** *TOR_LAB_ = AP x NO x NG x NT”*

• One major issue is double counting in Eq1. Tour operator revenue already includes taxes eventually paid to the government, thus you are counting them twice in Eq1, once as part of TOR and again as TX. Similar double counting occurs with guide license fees that are part of MPAR, occurring twice in Eq1, once in the TOR and again in the MPAR.

**Response:** Thank you for highlighting this. This is now changed and clarified in the manuscript. In the Maldives, all prices are subject to additional government corporate tax (TX, US$) and staff service charges (SSC, US$).

*Methods:*

“The government corporate tax (*TX,* US$) and the staff service charges (*SSC,* US$) in the Maldives in 2021 were 25% of the value of the services provided (i.e., 15% and 10%, respectively) [84]. Advertised prices in the Maldives are subject to both the tax (TX) and the staff service charge (SSC) [52,84].”

**Changed.** The tourist visitor entry fee (*EFv*) is deducted from Equation 2a and remains in Equation 4. There is now no double counting in Equation 1.

*Methods:*

“Importantly, the revenue generated from tourist visitor entry fees (*EF_V_*, US$) to Hanifaru MPA (*MPAR*) was deducted from *Equation 2a* and remained as revenue generated through MPA management (*Equation 4*).”

• TE is problematic with packages as opposed to paying for dives solely. Again, how did you divide up what visitors paid for packages into diving/viewing and accommodations, etc.

**Response:** Changed. The Tourist Expenses (*TE*) per guest for liveaboards was calculated by using 50% of the price per day.

*Methods:*

“Given that the price per day on a liveaboard includes food, beverages and accommodation, we assumed that the tourist expenses (*TE*) were 50% of the price per day (for each MRW dive, we calculated the tourist expenses (*TE*) using 50% of the price per guest on a liveaboard trip/nights per trip), calculated as US$189 for liveaboards. The cost benefit transfer ratios used were 13.265 for 5-star resorts, 7.233 for regular resorts, 2.703 for community islands and 2.586 for liveaboards.”

• Check all other equations for issues of double counting.

**Response:** Changed. We have confirmed that there is no additional double counting in any of the equations.

• Converting from Rufiya to US$ you used an exchange rate on a certain date instead of purchasing power parity that considers differences in the cost of living and is the economic approach necessary in this instance .

**Response:** Changed. Thank you for your guidance. We have updated the values that were converted from Maldivian Rufiyaa (MVR) to United States Dollar (US$) using the market exchange rate to now using the purchasing power parity (Maldives in 2021; 8.21066952; https://data.worldbank.org/indicator/PA.NUS.PPP). These values were the tourism and fisheries industries contribution to the GDP and the GDP Market Price of the Maldives.

These value updates of the GDP then impacted the contribution of MRW tourism to the GDP, which has also been updated through the manuscript.

*Abstract:*

“In 2021, manta ray tourism in the Maldives generated an estimated US$227.3 million, including US$39 million on manta ray focused diving and snorkelling excursions, and US$188.3 million in related tourist expenditure, representing 2.6% of the national Gross Domestic Product.”

*Introduction:*

“Tourism is the backbone of the Maldives economy, accounting for over a quarter of the nation’s US$8.9 billion Gross Domestic Product (GDP) in 2021 [49], with ~12% of the total labour force employed in resorts in 2022 [51].”

*Methods:*

“The national contribution of both the tourism [49,50] and fisheries industries [87] were compared to the GDP of the Maldives. All values used in this study were converted to US$ for consistency. Although the market exchange rate from Maldivian Rufiyaa (MVR) to United States Dollar (US$) on 01/01/2022 was 0.064826 [88], we used the purchasing power parity which was 8.2107 in the Maldives in 2021 [89]. While the market exchange rate highlights the industries’ role in the global market, the purchasing power parity-adjusted valuation underscores a higher domestic impact by considering the differences in the cost of living.”

*Results:*

“The combined revenue (TOR+TE) of MRW was estimated at US$227,298,377 and contributed an estimated 2.6% to the annual 2021 GDP purchasing power parity in the Maldives (Table 4).”

*Table 4:
“***Table 4*.*** **The Gross Domestic Product (GDP) in the Republic of Maldives and the share contribution of national industries including manta ray watching (MRW) tourism for 2008 – 2010, and 2019 – 2021**. The GDP and Tourism Contribution values were converted from Maldivian Rufiyaa (MVR) to United States Dollar (US$) for consistency using the Purchasing Power Parity which was 8.2107 in the Maldives in 2021 *[1]*.”

*Discussion:*

“The growth in revenue underscores the increasing economic significance of MRW tourism. Additionally, it mirrors that of the overall Maldives’ tourism sector which reported 286% growth in the tourism contribution to GDP over the same period (2008 = US$593.1 million and 2021 = US$2.3 billion) [49].”

“MRW tourism contributed substantially to the Maldives’ economy, representing 2.6% of its GDP in 2021, similar to that of the fishing and fish processing industry, which contributed 3.5% to the GDP in the same year [87].”

• Eq6 calculation of LV is not theoretically sound as it must consider the opportunity cost of natural capital, some sort of discount rate, or if omitted a discussion that justifies it.

**Response:** We thank the reviewer for this comment. We agree that the calculation in Eq. 6 requires clearer explanation and framing from an economic perspective.

Equation 6 estimates the cumulative tourism contribution of an individual manta ray by allocating total annual manta-related tourism revenue across the estimated population size and summing this annual contribution over the expected biological lifespan of the animal. This maintains comparability with the other elasmobranch valuations for the Maldives (Anderson et al. 2011) and globally (O’Malley et al., 2013).

To address the reviewer’s concern, we have revised the text to clarify the methodology and terminology, explicitly defining the lifetime value metric as gross and undiscounted. We have also added a discussion in the Caveats section explaining the concepts of discounting and opportunity cost and outlining why they were intentionally not included in this conservation-focused analysis. We believe these revisions provide the necessary economic context and ensure that the results are interpreted appropriately, without altering the objectives or conclusions of the study.

*Methods:*

“We adopted a non-discounted summation approach to estimate the cumulative tourism contribution attributed to an individual manta ray over its estimated lifespan. This method allocates total annual manta-related tourism revenue across the estimated population size and sums the resulting annual individual contribution over time. This approach was chosen to ensure direct comparability with previous elasmobranch tourism valuation studies [3,44]. We acknowledge that this method does not apply a discount rate to future revenues and does not incorporate the opportunity cost of natural capital. Consequently, the resulting estimates represent an undiscounted cumulative gross tourism contribution per individual, rather than a net present value or a full economic valuation.”

*Results:*

“The lifetime values (*LV*) are undiscounted cumulative gross tourism contributions per individual over an assumed 40-year biological lifespan. These were calculated using the atoll-specific generated revenue; for *M. birostris* using revenue from Fuvahmulah (*TOR_AS_* = US$705,036 and *TE_AS_* = US$1,575,327), and for *M. alfredi* using the revenue from the other regions (*TOR_AS_* = US$38,322,660 and *TE_AS_* = US$186,695,354). An individual *M. alfredi* was estimated to be worth US$2,571,634, compared to a value of US$91,215 for *M. birostris* during their lifetime (Fig 4).”

*Discussion:*

*“*When the current population estimate for *M. alfredi* (n=3,500) in the Maldives is considered to be interacting with the tourism industry over their ~40-year lifespan, the population would generate approximately US$8.6 billion (assuming real discount rate of 5%; see [4]) in undiscounted cumulative gross tourism contributions.”

“To calculate the lifetime values (*LV*) of both species, we did not apply a discount rate to account for the time value of money, whereby revenue generated in the future is typically considered less valuable than revenue realised in the present [122]. We also did not incorporate the opportunity cost of natural capital, defined as the alternative economic uses foregone by conserving manta rays for tourism rather than exploiting them for other purposes [123]. As a result, the lifetime values reported here should be interpreted as undiscounted cumulative gross tourism contributions per individual over an assumed 40-year biological lifespan [90], expressed in constant dollars. These values do not represent net present values or comprehensive economic valuations of manta rays as natural capital. While this approach may overstate the theoretical net present value, it remains a robust and pragmatic metric for conservation advocacy, effectively illustrating the scale of long-term economic loss associated with the removal of a single individual from the population [3,44].”

**REFERENCES**

Anderson RC, Adam MS, Kitchen-Wheeler A-M, Stevens G. Extent and Economic Value of Manta Ray Watching in Maldives. Tour Mar Environ. 2011;7: 15–27. doi:10.3727/154427310X12826772784793

O’Malley MP, Lee-Brooks K, Medd HB. The Global Economic Impact of Manta Ray Watching Tourism. Thrush S, editor. PLoS ONE. 2013;8: e65051. doi:10.1371/journal.pone.0065051

**3. Have the authors made all the data underlying the findings in their manuscript fully available?**
Not yet. The authors should specify what data archive will host the data. Usually, it is better practice to publish the data in a data depository, providing the DOI to reviewers.

**Response:** Thank you for the advice. Please find the data repository through Zenodo using DOI [10.5281/zenodo.18252206](https://doi.org/10.5281/zenodo.18252206)

**4. Is the manuscript presented in an intelligible fashion and written in standard English?**
Well written manuscript. The following are minor suggestions to improve clarity and flow.
• Check spelling of the short title: economic or economy

**Response:** Changed. Thank you.

Short title is now “Blue economy of manta ray watching in the Maldives”.

• Abstract: second to last sentence, eliminate “were”. Language is a bit pretentious and inflated.

**Response:** Changed. We have replaced “were” to “are”.

• Consider “charismatic” instead of “flagship species”. Animals are not manufactured assets, such as ships.

**Response:** Changed. We have updated the manuscript using “charismatic species” instead of flagship species. However, we would like this opportunity to suggest that manta rays do meet the necessary criteria to be classified as a flagship species (as discussed only in the *Discussion*):

*Introduction:*

“Ongoing management and monitoring of protected natural resources represents a significant financial commitment for governments, encompassing staff salaries, governance structure, and physical facilities [6,13]. To maximize conservation outcomes within constrained budgets, focusing efforts on charismatic species has emerged as a globally effective strategy for biodiversity conservation [14,15]. The selection of a species to serve as a conservation lead must be well-balanced and consider not only the intrinsic and ecological aspects (e.g., risk of extinction), but also the cultural, social and economic context of the region where the species will be promoted [16,17].”

“We hope that these findings will emphasise the national importance of an industry built upon charismatic species, providing the empirical evidence needed to support the continuation of ray sanctuaries, greater investment in MPA management, and manta ray conservation.”

*Discussion:*

“Recognising the value of charismatic species as the focus for wildlife tourism is becoming increasingly widespread [2–4,11,44,112].”

“Charismatic species, such as manta rays, serve as the biological embodiment of this natural goodness, providing the motivation for conservation actions that ultimately support broader food security and climate stability [118].”

“The growth in MRW tourism highlights the necessity for updated regional and global assessments to support the case for conservation. By demonstrating the immense economic and social value of these animals, such valuations promote non-consumptive resource use and aid the development of region-specific management. In the face of global population declines [71,97,124–129], this study suggests that manta rays fulfill the necessary criteria to be classified as a flagship species for the Maldives. Their high intrinsic, social and economic importance–coupled with their cultural significance to local communities–positions them as an ideal biological anchor for broader conservation efforts. Protecting such species not only conserves biodiversity but also fosters community engagement in conservation efforts, leveraging their cultural and economic value to motivate local stewardship. MRW tourism has the potential to continue developing into a long-term profitable and sustainable industry, resulting in sustainable livelihoods and business revenue for future generations in the years to come.”

• Change “economic evaluation” to “economic valuation”. You are not comparing economic performance against some sort of evaluation metric or a standard but instead you quantify values, thus, use “economic valuation.” Evaluation is a different social science method.

**Response:** Changed. Thank you for highlighting this. We have changed “economic evaluation” and simply “evaluation” to “economic valuation” or “valuation” throughout the document, including the *Abstract* and *Discussion*.

• Many instances of the use of “this” without actually naming what this is, specifically this followed by an object. Search and replace any “this”, and either eliminate or name what “this” is. I can read that mostly you refer to the prior sentence, but your writing becomes so much more clear if you synthesize the prior sentence using a term. L 54 for example, instead of “Despite this” write: “Despite the public intent to conserve, policy makers … “

**Response:** Changed. Good point - thanks for your suggestion that has improved the grammar of our manuscript. We have changed instances where we use “this” and replaced it with a reference to the topic discussed.

**5. Additional comments:**
Introduction
• Usually, intros are 2 double-spaced pages. Consider condensing significantly, perhaps to less than half the current length. Be concise on how your research fits into the broader literature. Perhaps, insert a subheading for manta ray ecology. Unless you compare the economic numbers from the literature to your results, there isn’t much need to present them all here.

**Response:** Updated. Thanks for your suggestion. We have removed 25% of the *Introduction* and used subheadings for manta ray ecology. We have also moved the economic numbers from the *Introduction* to the *Discussion*.

“Wildlife tourism can support both economic growth and the conservation of natural resources and ecosystems [1–4]. Categorised as a cultural ecosystem service, wildlife viewing provides essential non-material benefits, including recreational opportunities, aesthetic and spiritual experiences, learning and cultural enrichment [5]. These services are predicated on a shared valuation of the intrinsic beauty of wild species, which motivates tourists to visit and incentivises local communities to protect these natural assets [5]. However, policymakers are frequently challenged by the need to balance the requirements of multiple stakeholders competing for limited environmental resources [6]. Highlighting the socio-economic profitability of a well-managed wildlife tourism industry can help strengthen the case for protecting threatened species and ecologically important habitats by demonstrating their tangible value compared to extractive industries [7–9]. In successful frameworks, visiting tourists benefit from enhanced wellbeing and education [5,10], while local communities gain economic stability through employment and diversified income streams [9], which can encourage greater conservation efforts by both operators and visitors. Ultimately, these interactions create a powerful incentive for governments to establish robust protection measures and secure long-term stakeholder support [11,12].

Ongoing management and monitoring of protected natural resources represent a significant financial commitment for governments, encompassing staff salaries, governance structures, and physical facilities [6,13]. To maximize conservation outcomes within constrained budgets, focusing efforts on charismatic species has emerged as a globally effective strategy for biodiversity conservation [14,15]. The selection of a species to serve as a conservation lead must be well-balanced and consider not only the intrinsic and ecological aspects (e.g., risk of extinction), but also the cultural, social, and economic context of the region where the species will be promoted [16,17]. As such, marine tourism focusing on a charismatic species can make important contributions to biodiversity conservation and provide an “umbrella” of protection for lesser-known species [15]. This sector has expanded rapidly since the 1980s [1,11,18,19], generating direct revenue for operators and indirect financial flows for governments and local businesses [4,20–22]. In remote locations with few alternatives, well-managed megafauna tourism provides a critical pathway for communities to utilise their natural resources sustainably [19,23].

Shark and ray watching is a rapidly growing area of marine wildlife tourism, with activities established in at least 42 countries, primarily within low- to middle-income nations, including island nations [3,8,24,25]. Economic valuations consistently demonstrate that the tourism value of focal species can far exceed their value in extractive fisheries [3,24,26]. For instance, Anderson & Ahmed [27] in 1993 estimated the lifetime value of a single live shark in the Republic of Maldives was US$33,500, contrasted with US$32 for a landed shark. Despite the economic potential, coral-reef associated sharks and rays are among the most vulnerable marine taxa; nearly 59% of these species are threatened with extinction according to the International Union for Conservation of Nature (IUCN) Red List of Threatened Species, driven largely by overfishing and exacerbated by habitat degradation and climate change [28,29].

In regions where tourism infrastructure exists, shark and ray tourism offers a viable alternative to extractive practices. Currently, 29 countries have established shark and ray sanctuaries, which typically prohibit the commercial fishing, trade, possession, or sale of these taxa within their Exclusive Economic Zones [30–32]. These sanctuaries are often located in small-island nations across the Pacific Ocean, Caribbean Sea, and Indian Ocean [32], where reliance on marine resources is high [33] but enforcement capacity may be limited [34]. Recognising the economic importance of these species, the Maldives’ government implemented a national shark sanctuary in 2010 [35,36]. While rays and skates were first protected from the export trade in 1995/6, comprehensive legislation introduced in 2014 made it illegal to catch, kill, or harm all rays and skates, effectively making the Maldives a combined shark and ray sanctuary [36]. This legislative shift ended a century-long targeted shark (*miyaru* in the Maldivian Dhivehi language) fishery that utilised gillnets and longlines to harvest juvenile and adult individuals from 27 species for fins, meat, skin, liver oil, and jaws [37]. Unlike specialised offshore fisheries in other regions that target pelagic species in open water [38], the Maldivian shark fishery was a localised, multi-species operation that directly exploited coastal and reef-associated habitats shared by the burgeoning tourism industry [27,37] . These fisheries historically targeted many species that are now central to the Maldives’ tourism appeal, including whale sharks (*Rhincodon typus*), whitetip reef sharks (*Triaenodon obesus*), blacktip reef sharks (*Carcharhinus melanopterus*), grey reef sharks (*Carcharhinus amblyrhynchos*), scalloped hammerhead sharks (*Sphyrna lewini*), and tiger sharks (*Galeocerdo cuvier*) [27,37,39,40].

**Manta ray ecology and tourism**

Manta rays (*en madi* in Dhivehi) are zooplanktivorous giants found throughout tropical and subtropical oceans [41]. They aggregate seasonally at predictable locations that facilitate socialising and key life history functions including feeding, courtship and mating, predator avoidance, cleaning, and thermoregulation [42]. The predictable and reliable nature of these aggregations makes manta ray watching (MRW) a highly sought-after tourist activity [43–45] involving recreational diving, snorkelling, and boat-based observations of these animals in the wild [3]. The high value of these encounters is particularly evident in the Maldives, where tourists are willing to pay more for manta ray encounters than for those with sea turtles and reef sharks [46]. By 2013, the global MRW industry was valued at US$140 million per year (i.e., tour operator revenue and tourist expenses), across >25 countries, with the Maldives identified as the world’s leading destination with 101 MRW sites and 157,000 annual dives and snorkels [3].

The Maldives has successfully leveraged its marine biodiversity as a primary draw for international visitors since 1972 [47,48]. Tourist arrivals have grown consistently, reaching over 1.3 million in 2021 [49,50]. Tourism is the backbone of the Maldives economy, accounting for over a quarter of the nation’s US$8.9 billion Gross Domestic Product (GDP) in 2021 [49], with ~12% of the total labour force employed in resorts in 2022 [51]. Manta ray and shark watching are especially important components of the dive tourism industry [39,44,52].

However, this heavy economic dependence makes the nation acutely vulnerable to the climate crisis, habitat degradation, and overfishing [48]. While tourist arrival trends are often influenced by a complex interplay of political stability, global commodity prices, and natural disasters, the Maldives’ ‘brand’ is fundamentally built on its idyllic natural environment and rich biodiversity of marine life [48]. Consequently, any declines in the population health of charismatic marine species such as sharks and rays poses a direct threat to national economic stability [9,18,39,44,53]. These threats are compounded by historical precedents of resource depletion; for example, unsustainable shark fishing during the early 1990s led to declining shark numbers at popular dive sites, resulting in documented economic losses in the dive-tourism industry [9,43]. Notably, unlike sharks, manta rays have never been commercially targeted by Maldivian fisheries, providing a unique baseline for studying a relatively ‘pristine’ population [54].

The 26 atolls of the Maldives support the world’s largest known population of reef manta rays (*Mobula alfredi*), estimated to be 3,500 [55,56]. Mark recapture data of ~100,000 sightings recorded since 1989, and consistently since 2003, has identified over 6,200 unique individuals, though models suggest mortality rates need to be accounted for [55]. Additionally, the Maldives hosts a population of oceanic manta rays (*M. birostris*; at least for some of the year), with estimates suggesting an abundance of at least one order of magnitude greater than the ~1,000 individuals currently identified [57]. These populations aggregate at 48 known key aggregation sites (>100 sightings) [58], most notably the Hanifaru Marine Protected Area (MPA) in Baa Atoll, which serves as a critical foraging site [58]. Their distribution is influenced by the South Asian Monsoon; *M. alfredi* typically traverses the atolls from west to east as the monsoon shifts, ensuring year-round sightings [44,54,58]. Conversely, *M. birostris* are typically encountered in the south of the Maldives in Gnaviyani (referred to as Fuvahmulah hereafter) during the transition of the monsoons [57].

In 2008, the MRW industry in the Maldives was valued at US$15.5 million annually [3,44], though this figure was limited to direct revenue and did not account for broader socio-economic flow-ons, such as employee wages, government taxation, and secondary supply-chain benefits. Given the substantial growth in tourism over the last decade, the contemporary scale of this reliance remains largely unquantified. Both species of manta rays, *M. birostris* and *M. alfredi,* are globally threatened (listed as Endangered and Vulnerable respectively on the IUCN Red List) due to conservative life history traits, including late maturation and low fecundity [59–65]. Their recovery capacity is among the slowest of all elasmobranchs [60,66], making them highly susceptible to anthropogenic pressures [42,67,68]. Even within protected waters, poorly regulated tourism can lead to habitat destruction, behavioural disruption, and sub-lethal injuries [36,69–71]. Consequently, accurately estimating the socio-economic and intrinsic value of these species is vital for balancing the interests of tourism, fisheries, and coastal development [36].

In this study, we present the first national estimate of the direct economic benefits of the MRW tourism industry in the Maldives. We extend upon previous valuations (i.e., 1997, 2011 and 2013 [3,44,46]) by utilising surveys and data mining targeting tour operators, tourists, and government agencies. We aim to: (1) quantify the economic benefits across the business, government, and community sectors; (2) evaluate the size, scope, impact, and management of the industry; (3) estimate the lifetime economic value of individual *M. alfredi* and *M. birostris*; and (4) analyse the intrinsic value of manta rays to local communities. We hope that these findings will emphasise the national importance of an industry built upon charismatic species, providing the empirical evidence needed to support the continuation of ray sanctuaries, greater investment in MPA management, and manta ray conservation.”

• L163: unclear what negative pressure. From development or unenforced viewing regulations or some other factor.

**Response:** Updated. We have reworded this section to improve clarification.

“Their recovery capacity is among the slowest of all elasmobranchs [60,66], making them highly susceptible to anthropogenic pressures [42,67,68]. Even within protected waters, poorly regulated tourism can lead to habitat destruction, behavioural disruption, and sub-lethal injuries [36,69–71].”

Methods
• The first section belongs into the intro. This is background material, not methods. Instead, the methods section should start with your study design.

**Response:** Changed. Thanks for the suggestion, we have removed the Study Site section, with the *Methods* now starting with Study Design. The information on the manta ray distribution has been moved to the *Introduction*.

*Introduction:*

“Their distribution is influenced by the South Asian Monsoon; *M. alfredi* typically traverses the atolls from west to east as the monsoon shifts, ensuring year-round sightings [44,54,58]. Conversely, *M. birostris* are typically encountered in the south of the Maldives in Gnaviyani (referred to as Fuvahmulah hereafter) during the transition of the monsoons [57].”

• What was your sample size and response rate for the pilot?

**Response:** Changed. We have included the sample size and response rate into the *Methods*:

“Prior to data collection, the survey was first piloted on experts in the industry with feedback incorporated into the survey design (n=10, 100% response rate).”

• L262, comparing response rates to other studies belongs into the discussion.

**Response:** Changed. Removed the comparison in the *Methods* and added it to the caveats in the *Discussion*.

*Methods:*

“All 538 tour operators in the Maldives were contacted and offered the chance to voluntarily participate in the survey, with 106 operators responding (20% response rate).”

*Discussion:*

“The ~20% response rate obtained in our study is comparable to participation levels in previous wildlife tourism assessments [38,52].”

• L366: United States not “stated”

**Response:** Changed.

• L387-390: how did you estimate this?

**Response:** Updated information in the manuscript explaining more clearly how liveaboard profit in Fuvahmulah Atoll was calculated.

*Methods:*

“For *M. birostris*, the revenue from the tour operators was generated in Fuvahmulah only, while the revenue for *M. alfredi* corresponds to all the other regions they are sighted seasonally. Two liveaboard tour operators who completed the survey (*n*=8) highlighted that they visited Fuvahmulah Atoll in the Northeast Monsoon. Also in the same season, Manta Trust’s *Maldives Manta Conservation Programme* long-term monitoring database revealed regular presence of liveaboards in routine surveys [55,91]. We estimated that 4% of the revenue generated (*TOR_AS_ + TE_AS_*) from liveaboards was from time spent in Fuvahmulah and the other 96% of revenue was from the remaining regions, based on the responses on visitation frequency from eight tour operators.”

• L399-404: how was this “text mining” done? A careful analysis of text is usually done following qualitative data analysis that includes determining coding categories before analyzing text. This approach can then be used to carefully assess motivations. Word clouds are problematic and should not be used in academic publications as there is no analysis, no rigor. They are good for presentations and data exploration.

**Response:** Updated. Thank you for your feedback. We have removed the word clouds and kept the word count analysis only. This has been updated in the *Methods*, *Results* and removed Figure 6 from the manuscript.

“Word count analysis was used to show the most frequently used words to describe the importance of MRW tourism by survey respondents. Words count analysis and the removal of stop words was done using the ‘tidytext’ R package [92]. Some of the words used in the questions were also removed pre-analysis e.g., “manta”, “mantas”, “manta ray”, “Maldives”.”

## Reviewer #2

**5. Review Comments to the Author

Please use the space provided to explain your answers to the questions above. You may also include additional comments for the author, including concerns about dual publication, research ethics, or publication ethics. (Please upload your review as an attachment if it exceeds 20,000 characters)**

**Review of PONE-D-25-29750**

This MS is well written, and the authors successfully highlight its value. First of all, as an updated study of the economic value of Maldives MRW after more than a decade is very important for future management. This study also highlights the discrepancy in staff salary revenue between Maldivian and expatriate staff, as well as the need for more stable funding that supports in-country conservation from tourism. Further, this study is also very important for backing arguments against unsustainable fishing practices (such as the recent plan to legalise shark fishing in the Maldives in 2024) and any future threats to biodiversity. Minor improvements are needed, including elaboration on fisheries and cultural significance.

**Response:** Thank you for your positive feedback and important revisions.

123: Could be a separate paragraph about threats and challenges.

**Response:** Changed. Thanks for the suggestion, we have made threats and challenges a separate paragraph from Maldives economics in the *Introduction*:

“Tourism is the backbone of the Maldives economy, accounting for over a quarter of the nation’s US$8.9 billion Gross Domestic Product (GDP) in 2021 [49], with ~12% of the total labour force employed in resorts in 2022 [51]. Manta ray and shark watching are especially important components of the dive tourism industry [39,44,52].

However, this heavy economic dependence makes the nation acutely vulnerable to the climate crisis, habitat degradation, and overfishing [48].”

97-100 and 131-132:

Please explain the magnitude of shark fishing in the Maldives. Is it mainly bycatch or targeted? And when did it start?

What species of shark were extracted? If it is a specialised shark fishery, the fishers tend to extract specific species and sizes to maximise the catch (fins are usually the main body parts in targeted shark fisheries) such as adult thresher, silky shark, hammerhead, etc and specialised fishers usually using longlines that mostly operates in open water instead of the reefs /coast where marine tourism (such as diving) took place. If the sharks at dive sites, such as reef and tiger sharks (which targeted shark fishers do not prioritise), are declining, it is not necessarily because of specialised shark fisheries. See Mustika et al. (2018) <https://doi.org/10.3389/fmars.2020.00261> for more information. Also, declining visit numbers in a country’s tourism might be a combination of several factors such as political instability, rising commodity prices, and natural disasters.

**Response:** Thank you for the suggestion, we have incorporated the magnitude of the shark fishing industry of the Maldives into a paragraph of the *Introduction* and the visitor numbers into another:

“While rays and skates were first protected from the export trade in 1995/6, comprehensive legislation introduced in 2014 made it illegal to catch, kill, or harm all rays and skates, effectively making the Maldives a combined shark and ray sanctuary [36]. This legislative shift ended a century-long targeted shark (*miyaru* in the Maldivian Dhivehi language) fishery that utilised gillnets and longlines to harvest juvenile and adult individuals from 27 species for fins, meat, skin, liver oil, and jaws [37]. Unlike specialised offshore fisheries in other regions that target pelagic species in open water [38], the Maldivian shark fishery was a localised, multi-species operation that directly exploited coastal and reef-associated habitats shared by the burgeoning tourism industry [27,37]. These fisheries historically targeted many species that are now central to the Maldives’ tourism appeal, including whale sharks (*Rhincodon typus*), whitetip reef sharks (*Triaenodon obesus*), blacktip reef sharks (*Carcharhinus melanopterus*), grey reef sharks (*Carcharhinus amblyrhynchos*), scalloped hammerhead sharks (*Sphyrna lewini*), and tiger sharks (*Galeocerdo cuvier*) [27,37,39,40].”

“However, this heavy economic dependence makes the nation acutely vulnerable to the climate crisis, habitat degradation, and overfishing [48]. While tourist arrival trends are often influenced by a complex interplay of political stability, global commodity prices, and natural disasters, the Maldives’ ‘brand’ is fundamentally built on its idyllic natural environment and rich biodiversity of marine life [48].”

136-137*:* 3,500 individuals are from mark-recapture analysis; what about the 6,200 individuals? Please elaborate to avoid confusion.

**Response:** Changed. The *M. alfredi* populations data recording started in 1989, with consistent data collection since 2003. *Mobula alfredi* live for several decades, but many obviously die as a result of mortality events earlier than this. Therefore, although the total number of individuals recorded now stands at over 6,000, it is estimated that ~41% of these rays are now estimated to have died. This population estimate manuscript is currently in preparation (Froman & Stevens In Prep.). It is more accurate to base our numbers in this study on the actual population and not the total number of individuals recorded since the study began, similar to other studies (see Anderson et al. 2011, O’Malley et al. 2013).

*Introduction:*

“The 26 atolls of the Maldives support the world’s largest known population of reef manta rays (*Mobula alfredi*), estimated to be 3,500 [55,56]. Mark recapture data of ~100,000 sightings recorded since 1989, and consistently since 2003, has identified over 6,200 unique individuals, though models suggest mortality rates need to be accounted for [55].”

**REFERENCES**

Anderson RC, Adam MS, Kitchen-Wheeler A-M, Stevens G. Extent and Economic Value of Manta Ray Watching in Maldives. Tour Mar Environ. 2011;7: 15–27. doi:10.3727/154427310X12826772784793

Froman N, Stevens GMW. Population size and structure of reef manta rays, Mobula alfredi, in the Maldives. Forthcoming.

O’Malley MP, Lee-Brooks K, Medd HB. The Global Economic Impact of Manta Ray Watching Tourism. Thrush S, editor. PLoS ONE. 2013;8: e65051. doi:10.1371/journal.pone.0065051

298-304: Liveaboard trips’ diving numbers are hard to quantify, since the number of dives at manta sites is determined by customer requests, and some divers may dive more than others. Are there any other non-manta-ray diving sites with significant visitation?

**Response:** Updated. We have clarified in the manuscript that the mean number of MRW diving opportunities (NO) was used to quantify manta ray diving on liveaboards. There are many non-manta ray focused dive and snorkel sites located across the Maldives. As well as liveaboards that run trips during both monsoon seasons, meaning MRW sites are not active year-round.

*Methods*:

*“TOR_LAB_* (US$) was calculated as the product of the value of the activity price (*AP*, US$), the number of opportunities to snorkel and dive with manta rays (*NO*, unitless), the number of guests per trip (*NG*, unitless), and the number of trips per year in 2021 (*NT*, year^-1^; *Equation 2b*). As *TOR_LAB_* already accounted for tourist expenses (*TE*) as they are sold as a package, we estimated that only half of the price per trip (50%) was used to cover diving expenses and thus was used to calculate the activity price (*AP*) using the overall number of dives offered per liveaboard trip. We then used the activity price (*AP*) and the number of opportunities (*NO*) to snorkel and dive with manta rays to calculate the price per MRW dive. Where values for the activity price (*AP*), the number of guests (*NG*), the number of trips (*NT*), and the number of opportunities (*NO*) were missing from tour operators (e.g., non-responding operators), a mean value was assigned.”

***“Equation 2b.*** *TOR_LAB_ = AP x NO x NG x NT”*

371: For the estimated lifespan, I suppose authors use the whole lifespan, as used in many references.

I think it would be interesting to consider what age/size manta rays are occupied by or seen at the diving sites as a marine tourism highlight? Are the younger, smaller manta rays seen often at the main dive sites? Do they have separate aggregation sites before they reach maturity (about 8 years)?

See Setyawan et. al. 2022a <https://doi.org/10.3389/fmars.2022.815094>

**Response:** Changed. Thanks for the suggestion as you highlight a really good point. We have added a sentence in the *Methods* specifically about juvenile vs adult visitation at both dive and snorkel sites, however going into more detail is beyond the scope of this study.

“The population of *M. alfredi* was assumed to be 3,500 individuals [55,56] and for *M. birostris* 1,000 individuals [55]. Both populations are a mixture of adult and juvenile (reaching maturity at 8–17 years age [65]), with mostly adults visiting diving sites and a mixture of both at snorkelling sites [55,57].

385-386: While *M. alfredi* move locally between seasons, and *M. birostris* MRW in Fuvahmulah (Table 1) are active only in the NE monsoon, do we have any information (for example, from tagging studies) on where they are for the rest of the year?

**Response:** Such an interesting research question that is currently under review by S. Hilbourne at The Manta Trust. They successfully deployed three satellite tags in 2025, with plans to deploy additional tags in early 2026. The manuscript is not yet in preparation.

387-390: With only eight responses from 13% of completed surveys among liveaboards, I will be cautious in estimating revenue (4%) and state that liveaboards mostly visit Fuvahmulah. If there is more information/reference regarding this, please add it to the MS.

**Response:** Updated. We have continued to use the 4% but with further explanation and added an additional reference.

*Methods*:

“For *M. birostris*, the revenue from the tour operators was generated in Fuvahmulah only, while the revenue for *M. alfredi* corresponds to all the other regions they are sighted seasonally. Two liveaboard tour operators who completed the survey (*n*=8) highlighted that they visited Fuvahmulah Atoll in the Northeast Monsoon. Also in the same season, Manta Trust’s *Maldives Manta Conservation Programme* long-term monitoring database revealed regular presence of liveaboards in routine surveys [55,91]. We estimated that 4% of the revenue generated (*TOR_AS_ + TE_AS_*) from liveaboards was from time spent in Fuvahmulah and the other 96% of revenue was from the remaining regions, based on the responses on visitation frequency from eight tour operators.”

419: Does the number of boats per trip mean the number of boats sighted at a time in a particular site? Please elaborate.

**Response:** Changed. We have clarified what the number of boats per trip means in the *Results* of the manuscript.

“Hanifaru MPA was the most important MRW site in terms of number of tourists counted at the time of visitation during a trip (*n*=52.6 ± 6.1 Standard Error (SE)), number of boats counted during a trip (*n*=8.8 ± 1.2 SE), and the number of manta rays sighted during a trip (snorkelling trips only) (*n*=32.3 ± 4.0 SE).”

420: How to define a trip? For manta rays sighted per trip, is it more accurate if it’s manta rays per dive? And do you have information on how many dives per trip? Please state in the MS.

**Response:** Changed. In this study, we use the term "trip" as a standardised unit of effort encompassing both scuba diving and snorkelling sessions. Specifically, a trip is defined as a single dive or snorkel session. For example, a "two-tank dive" is accounted for as two separate trips. We have clarified the definition of a "trip" in the Study Design of the *Methods* and the *Supporting information* (S1 Table), thus clarifying what the number of mantas per trip / per dive means in the *Results* of the manuscript.

*Methods:*

“MRW guests are those who participated in a MRW trip, which may include individuals who participated in multiple trips (S1 Table). A MRW trip is a single dive or snorkel session on a vessel. For example, a two-tank dive is accounted for as two separate trips.”

*Results:*

“Hanifaru MPA was the most important MRW site in terms of number of tourists counted at the time of visitation during a trip (*n*=52.6 ± 6.1 Standard Error (SE)), number of boats counted during a trip (*n*=8.8 ± 1.2 SE), and the number of manta rays sighted during a trip (snorkelling trips only) (*n*=32.3 ± 4.0 SE).”

*Supporting information:*

“Trip: A trip is a single dive or snorkel session with manta rays taken by guests on a vessel. For example, a two-tank dive counts as two trips.”

421: “Encounters with *M. birostris* were only promoted in Fuvahmulah”, already mentioned in the previous section. Consider removing or combining it with the previous sentence.

**Response:** Changed. Thank you. We have removed the repetition.

Removed: “Encounters with *M. birostris* were only promoted in Fuvahmulah”

523-525: Interesting point. Do you have more specific information about manta rays’ cultural value within the community? Any cultural products, such as myths, legends, and Folklore, as found in many indigenous societies around the world? This value, if communicated well, can strengthen a sense of identity and support conservation.

**Response:** Changed. Thank you for such an insightful perspective. In the survey we asked the tour operators: “2. Why/why-not do you consider manta rays to be important to local communities in the Maldives”. The most detail we received was that manta rays are culturally significant, but not the specifics of why. However, we agree this is something that should be asked in future socio-economic studies. We have added this recommendation to the *Discussion*:

“For future studies, we recommend asking an additional survey question directed towards cultural significance of the target species within the indigenous society, such as myths, legends and Folklore. Such information has the potential to demonstrate a strong cultural connection and in turn support conservation.”

537: You can also explain the response on “how manta rays ranked among marine life visitors wanted to see” in a sentence instead of in Figure 5.

**Response:** Changed. Thank you for the suggestion. We have removed “Rank among marine life” from Figure 5, and have elaborated only in the *Results* text:

“All tour operators ranked manta rays in the top five sea life that visitors most wanted to see (*n*=100) with 28% ranking them as the top attraction, 47% ranking them in their top two, 19% in their top three and 6% in their top four.”

**“Fig 5. The perceived socio-economic importance of manta ray watching tourism to tour operators (proportion) in the Republic of Maldives.** These responses were via survey questions about the nature of the value e.g., to their business, the community, and as a tool for education and conservation for guests.”

597: You can add more examples from regions where manta rays or other elasmobranch species are valued as a marine tourism spectacle but also facing threats of unsustainable fishing (e.g., Indonesia, Philippines). See O’Malley 2013 <https://journals.plos.org/plosone/article/file?id=10.1371/journal.pone.0065051&type=printable>.

Also, be mindful of stating that fisheries mortality contributes to the decline in elasmobranch species at dive sites, since many specialised elasmobranch fisheries target specific species and sizes, and operate offshore rather than in tourism spots, as I mentioned in the previous section.

When comparing MRW management across countries, it is helpful to include additional context on each country’s size and management to provide readers with more information. For example, the Raja Ampat region in Indonesia alone is a 40,000 km² Regency, with several manta ray dive sites and 51 manta-sighting sites, managed as a network of five MPAs by the local MPA Network Management Unit (BLUD), which might differ from the Maldives’ centralised MPA management.

See Setyawan 2022b <https://doi.org/10.3389/fmars.2022.1014791>

**Response:** Changed. Thank you for the suggestion. We have updated the *Discussion* to provide a more nuanced comparison of global MRW management. This includes acknowledging the spatial distinction between offshore elasmobranch fisheries and tourism sites, while noting the population-level impacts of the former. Furthermore, we have incorporated the suggested examples of Indonesia (Raja Ampat) and the Philippines to contrast the Maldives' centralised management with localised, regional MPA networks.

“The Maldives is a tuna fishing nation [96] and has an especially high dependence on the sustainable availability of marine resources for both food security and economic prosperity [33]. As a country that does not harvest its manta rays [36], but is reliant on both fisheries and tourism industries, the Maldives provides an example of how these two industries can co-exist when managed sustainably. This contrasts with regions such as Mozambique, where manta ray sightings in one tourism hub decreased by 92.5–99% over two decades, largely due to fisheries mortality [97]. While specialised elasmobranch fisheries often operate offshore and target specific size classes—meaning they do not always overlap spatially with tourism sites—the resulting population-level declines can eventually impact sighting rates at dive locations [3].

Similar tensions between tourism and unsustainable harvesting exist in other biodiversity hotspots like Indonesia and the Philippines [3]. These regions face the complex challenge of balancing lucrative elasmobranch tourism with active local fisheries. However, management scales and structures differ significantly across these locations. For instance, the Raja Ampat region in Indonesia encompasses a 40,000 km² Regency where a network of five MPAs is managed by a localised management unit [98]. This decentralised approach allows for regional oversight of its 51 known MRW sites, contrasting with the more centralised management framework of the Maldives’ MPA network. Understanding these diverse management models is critical for replicating the economic success of the Maldives in other regions facing similar threats.”

662: You might want to start with the negative impacts on manta rays. Or start the paragraph with “When tourism is unmanaged or is beyond the carrying capacity of an area, the industry is unsustainable and requires further regulation and enforcement”, and give examples of manta rays and cetaceans.

**Response:** Changed. This paragraph in the *Discussion* has been rearranged in a more logical order.

“When tourism is unmanaged or is beyond the carrying capacity of an area, the industry is unsustainable and requires further regulation and enforcement. In the Maldives, sublethal injuries [70] and tourist behaviour [69] negatively impact manta rays and lead to a possible reduction in reproductive fitness [56]. These impacts can become more pronounced during the climate crisis due to a reduced availability of the manta ray’s planktonic prey which is required to sustain reproductive health and overall fitness [62]. Other animal groups such as cetaceans have shown to react to tourist vessels with active avoidance strategies [105,106]. Such repeated disturbances even led to displacement from a preferred habitat and reduced fitness at the population level for bottlenose dolphins (Tursiops *sp.*) [105]. The projected reduction in reproductive fitness of manta rays with exposure to threats highlight the importance of safeguarding known foraging areas and other critical habitats. Despite the expansive nature of MRW activities in the Maldives, the visitation density at these numerous sites remains unquantified. Investigating the crucial metric of visitation density in the future is essential for robustly assessing and managing the potential negative impacts of tourism.”

724: Similar to lines 523-525, please elaborate more about “natural goodness”, which might be very interesting for the readers.

**Response:** Updated. The Intrinsic value of manta rays’ section in the *Discussion* has been updated to elaborate more on the natural goodness, and linking it more closely to our *Results*.

“Tour operators in the Maldives perceived manta rays to be important to the local community, emphasising their cultural significance beyond economic utility. Marine animals like whales have historically been recognised for their “natural goodness”–a concept describing the inherent value of a creature based on its ability to satisfy the necessities of its life form [117]. This recognition was a large driver of the “save the whales” movement, shifting public perception from whales as a commodity to beings with intrinsic rights to exist [117]. Our findings suggest a similar sentiment among Maldivian stakeholders; by describing manta rays through terms like “local” and “cultural significance,” respondents acknowledge the rays’ material goodness as a standard for evaluating human treatment of the marine environment.

This ethical foundation provides powerful grounds for conservation practices that transcend beyond financial justifications [117]. Charismatic species, such as manta rays, serve as the biological embodiment of this natural goodness, providing the motivation for conservation actions that ultimately support broader food security and climate stability [118]. As MRW tourism is reliant on the quality of the experience, it in turn promotes a healthy marine environment and engages the public and community in safeguarding the species’ ability to flourish. Consequently, the conservation of charismatic species has ripple effects, yielding widespread benefits for ecosystem health by enabling the conservation of intact environments, restoration of degraded ones, conserving biodiversity, and achieving sustainable development goals [14].”

**Why the paper is good**

This MS is well written, and the authors successfully highlight its value. First of all, as an updated study of the economic value of Maldives MRW after more than a decade is very important for future management. This study also highlights the discrepancy in staff salary revenue between Maldivian and expatriate staff, as well as the need for more stable funding that supports in-country conservation from tourism. Further, this study is also very important for backing arguments against unsustainable fishing practices (such as the recent plan to legalise shark fishing in the Maldives in 2024) and any future threats to biodiversity. Minor improvements are needed, including elaboration on fisheries and cultural significance.

**Response:** Thank you for your positive feedback and very helpful revisions!
