# Supplementary material for: Valuing conservation and natural wealth: The blue economy of manta ray watching in the Maldives
Source: PLoS One. 2026 Jun 1;21(6):e0326719. doi: 10.1371/journal.pone.0326719 (PMC13225372; doi:10.1371/journal.pone.0326719)
Supplement: Attachment — Submitted filename: Response_to_Reviewers_auresp_2.docx [file pone.0326719.s007.docx]

# PLOS ONE – minor revisions

**Contents of letter “Response to Reviewers”**

**Table of Contents**

[PLOS ONE – minor revisions	1](#_Toc225327193)

[Letter from Editor	1](#_Toc225327194)

[Reviewers Responses to Questions	3](#_Toc225327195)

[Reviewer Comments to the Author	4](#_Toc225327196)

## Letter from Editor

PONE-D-25-29750R1

Valuing conservation and natural wealth: The blue economy of manta ray watching in the Maldives

| **Date:** | Mar 06 2026 03:40AM |
| --- | --- |
| **To:** | "Hannah Maree Moloney" hannah.moloney@mantatrust.org;hannah.moloney@research.usc.edu.au |
| **From:** | "PLOS ONE" plosone@plos.org |
| **Subject:** | PLOS ONE Decision: Revision required [PONE-D-25-29750R1] |

PLOS One

Dear Dr. Moloney,

Thank you for submitting your manuscript to PLOS ONE. After careful consideration, we feel that it has merit but does not fully meet PLOS ONE’s publication criteria as it currently stands. Therefore, we invite you to submit a revised version of the manuscript that addresses the points raised during the review process.

Please submit your revised manuscript by Apr 20 2026 11:59PM. If you will need more time than this to complete your revisions, please reply to this message or contact the journal office at [plosone@plos.org](mailto:plosone@plos.org). When you're ready to submit your revision, log on to https://www.editorialmanager.com/pone/ and select the 'Submissions Needing Revision' folder to locate your manuscript file.

Please include the following items when submitting your revised manuscript:

- A letter that responds to each point raised by the academic editor and reviewer(s). You should upload this letter as a separate file labeled 'Response to Reviewers'.
- A marked-up copy of your manuscript that highlights changes made to the original version. You should upload this as a separate file labeled 'Revised Manuscript with Track Changes'.
- An unmarked version of your revised paper without tracked changes. You should upload this as a separate file labeled 'Manuscript'.

If you would like to make changes to your financial disclosure, please include your updated statement in your cover letter. Guidelines for resubmitting your figure files are available below the reviewer comments at the end of this letter.

If applicable, we recommend that you deposit your laboratory protocols in protocols.io to enhance the reproducibility of your results. Protocols.io assigns your protocol its own identifier (DOI) so that it can be cited independently in the future. For instructions see: <https://journals.plos.org/plosone/s/submission-guidelines#loc-laboratory-protocols>. Additionally, PLOS ONE offers an option for publishing peer-reviewed Lab Protocol articles, which describe protocols hosted on protocols.io. Read more information on sharing protocols at <https://plos.org/protocols?utm_medium=editorial-email&utm_source=authorletters&utm_campaign=protocols>.

We look forward to receiving your revised manuscript.

Kind regards,

Joel Harrison Gayford
Academic Editor
PLOS One

**Response:** Thank you for giving us the opportunity to resubmit our manuscript for a second round of revisions. We have addressed the feedback and provided point by point detailed responses below.

**Journal Requirements:**

If the reviewer comments include a recommendation to cite specific previously published works, please review and evaluate these publications to determine whether they are relevant and should be cited. There is no requirement to cite these works unless the editor has indicated otherwise. 

Please review your reference list to ensure that it is complete and correct. If you have cited papers that have been retracted, please include the rationale for doing so in the manuscript text, or remove these references and replace them with relevant current references. Any changes to the reference list should be mentioned in the rebuttal letter that accompanies your revised manuscript. If you need to cite a retracted article, indicate the article’s retracted status in the References list and also include a citation and full reference for the retraction notice.

## Reviewers Responses to Questions

**Comments to the Author**

1. If the authors have adequately addressed your comments raised in a previous round of review and you feel that this manuscript is now acceptable for publication, you may indicate that here to bypass the “Comments to the Author” section, enter your conflict of interest statement in the “Confidential to Editor” section, and submit your "Accept" recommendation.

Reviewer #1: All comments have been addressed

Reviewer #2: All comments have been addressed

2. Is the manuscript technically sound, and do the data support the conclusions?

The manuscript must describe a technically sound piece of scientific research with data that supports the conclusions. Experiments must have been conducted rigorously, with appropriate controls, replication, and sample sizes. The conclusions must be drawn appropriately based on the data presented.

Reviewer #1: Yes

Reviewer #2: Yes

3. Has the statistical analysis been performed appropriately and rigorously?

Reviewer #1: Yes

Reviewer #2: Yes

4. Have the authors made all data underlying the findings in their manuscript fully available?

The [PLOS Data policy](http://www.plosone.org/static/policies.action#sharing) requires authors to make all data underlying the findings described in their manuscript fully available without restriction, with rare exception (please refer to the Data Availability Statement in the manuscript PDF file). The data should be provided as part of the manuscript or its supporting information, or deposited to a public repository. For example, in addition to summary statistics, the data points behind means, medians and variance measures should be available. If there are restrictions on publicly sharing data—e.g. participant privacy or use of data from a third party—those must be specified.

Reviewer #1: Yes

Reviewer #2: Yes

5. Is the manuscript presented in an intelligible fashion and written in standard English?

PLOS ONE does not copyedit accepted manuscripts, so the language in submitted articles must be clear, correct, and unambiguous. Any typographical or grammatical errors should be corrected at revision, so please note any specific errors here.

Reviewer #1: Yes

Reviewer #2: Yes

**Response:** Thank you to both Reviewers for taking the time to ensure this manuscript is sound and appropriate for publication in PLOS ONE.

Reviewer Comments to the Author

Please use the space provided to explain your answers to the questions above. You may also include additional comments for the author, including concerns about dual publication, research ethics, or publication ethics. (Please upload your review as an attachment if it exceeds 20,000 characters)

Reviewer #1: I thank the authors for their continued and quite extensive efforts to improve this manuscript. I can accept the revisions and the paper should be published after some additional minor modifications, that I may suggest as follows:

**Response:** Thank you for your positive feedback, as well as your helpful revisions which have improved the manuscript.

• Figures are all low dpi, increase dpi to at least 300dpi, Fig 3 fonts need to be legible, Fig. 4 has missing axis and missing labels … what are you showing?

**Response:** Changed. All figures are 300 dpi, the font size on Fig 3 is now larger making it more legible, and Fig 4 now has labels (key and north arrow). We have not added axis labels as this figure is more of an infographic than a graph, however we agree that the contents of Fig 4 needed to be made clearer from the stand-alone figure. All files have been re-uploaded with increased resolution during the resubmission process.

**Fig 4. The lifetime value of individual manta rays (both species) as part of tourism calculated using the combined economic revenue from manta ray watching in the Republic of Maldives in 2021.** Tourism that focused on *Mobula alfredi* (reef manta rays) was identified in 18 administrative regions, compared to only one region for *M. birostris* (oceanic manta rays; Gnaviyani/Fuvahmulah). The geographical extent of manta ray tourism for each species is shown using coloured dotted rectangles.

• Lines 574-577. Explain the different methods used in the past and that you use and then give an honest assessment of your estimate describing factors contributing to over and underestimating the true value. It’s good that you cite numbers to validate growth but your estimate needs more discussion how it fits past estimates and how not. Also, check the statement in the quoted lines. To me it is a bit contradictory. Consider revising to eliminate the contradiction.

**Response:** Changed. Thank you for this suggestion. We have revised the opening paragraph of the *Discussion* to provide a transparent comparison of methodologies and a balanced assessment of our valuation. The statement that was in lines 574-577 has been revised and eliminates the contradiction.

*Discussion:*

“**Economic benefits of manta ray watching tourism**

We demonstrate that marine wildlife tourism provides immense value to local and national economies by generating revenue, supporting cultural heritage, and creating employment opportunities. Using the Maldives as a case study, this study provides an updated national valuation of the economic benefits of MRW tourism. We found that the 475,000 dive and snorkel trips taken by MRW guests in 2021 generated an estimated US$227.3 million, comprising US$39 million in revenue for tour operators and US$188.3 million in revenue of secondary tourist expenditures. These estimates represent a substantial increase from previous assessments in 1997 (US$7.8 million) and 2008 (US$8.1 million) [43,44]. When the industry was last valued at a national scale in 2011 (using 2008 data), it was estimated to generate US$15.5 million in combined revenue, including US$7.4 million in tourist expenditures derived from the 157,000 MRW trips [3].

While these estimates suggest significant expansion, direct comparisons are complicated by evolving methodologies. The 2011 study [3] utilised a site-density approach for MRW sites, whereas our study employed a high-resolution approach combining tour operator surveys and online data mining. Both estimates are inherently conservative, accounting only for quantifiable metrics; however, the 2011 estimate was limited by its focus on tourism density at known MRW sites, and the benefit transfer ratio used to calculate secondary tourist expenses did not account for pricing tiers between operator types (e.g., expenses at a luxury resorts vs at a community island) [3]. Further, our 2021 estimate may be influenced by broader market inflation and a more comprehensive capture of secondary supply chains that were previously unmonitored.

Despite these methodological differences, the 2021 value reflects a remarkable >380% growth in tour operator revenue over the past decade. The growth underscores the increasing economic significance of MRW tourism and mirrors the trajectory of the broader Maldivian tourism sector, which reported a 286% increase in GDP contribution over the same period (2008 = US$593.1 million and 2021 = US$2.3 billion) [49]. Consequently, while the different methods utilised over the last two decades prevent precise direct comparisons of absolute values, the overarching trend clearly establishes MRW tourism as a cornerstone of the national economy.”

The discussion section could benefit from a few suggested points:
- Discrepancy between foreign and Maldivian staff salaries, despite the latter most likely having better local knowledge. You could specifically point out the distribution of benefits between local and foreign staff with policy implications. Why are foreign guides needed? Could the government levy a tax for foreign guides and use that income to bolster local guides’ workforce development, education, etc. Also, note that the involvement of locals in conservation-oriented tourism such as manta ray guiding and promoting local employment in this sector with rising wages could significantly play into competing successfully against opportunities locals have in the illegal harvesting sector of the manta ray economy. Harvesters may have traditional knowledge that they could immediately deploy in the tourism sector if wages are better there.

**Response:** Changed. We have updated the employment section in the *Discussion* to incorporate these constructive suggestions regarding the socio-economic distribution of tourism benefits. While manta rays are not harvested in the Maldives, we argue that failing to provide competitive career trajectories for locals may result in the loss of invaluable human capital, specifically Traditional Ecological Knowledge.

Regarding the suggestion to discuss specific government levies or the reasons for foreign guide recruitment; we have respectfully omitted a detailed analysis of these policy mechanisms. While foreign guides are commonly hired to cover specific skill gaps or service requirements (such as specialised language skills or academic background in marine biology), we feel that a deep dive into labour-market structures or specific recruitment requirements is beyond the scope of this study’s primary economic focus. We have instead focused the discussion on the broader economic necessity of upskilling and equitable benefit retention to support long-term conservation.

*Discussion*:

“Both the Maldivian economy and the livelihood of its people are largely dependent on marine resources [97]. This study provides evidence for the flow-on socio-economic impacts of manta ray tourism in the Maldives. Almost 4,000 staff were employed by tour operators to work directly on MRW diving and snorkelling tourism activities, generating US$27 million per year in staff salary revenue. Despite the 2,602 Maldivian staff representing 67% of the MRW tourism workforce, they only received 55% of the distributed staff salary within the industry. A similar discrepancy was observed in reef shark-diving tourism in 2016, where Maldivians represented 55% of the workforce but received 50% of the distributed staff salary [52]. The wage gap likely stems from the disproportionate employment of Maldivian nationals in lower-paid operational roles, such as divemaster, equipment maintenance, or boat operations, while expatriates more frequently occupy senior management and instructional positions, a pattern observed in many Global South tourism industries [98,99].

While some operators in the Maldives are already implementing initiatives to further address these disparities, encouraging the widespread adoption of a structured approach toward localisation remains vital. Beyond fostering individual career progression, these initiatives offer broader benefits, including enhanced local economic development, long-term economic sustainability, and reduced recruitment costs [98,100]. Formalising and rewarding the high-value traditional ecological knowledge possessed by Maldivian guides with competitive, senior-level wages is essential for ensuring that marine tourism offers a lucrative and prestigious career path. Maldivians often possess knowledge regarding manta ray aggregations and oceanographic patterns that is indispensable for high-quality guest experiences. If the tourism sector fails to provide a career trajectory that competes with other high-paying industries, this human capital may be lost to sectors that do not contribute to the blue economy or conservation. By incentivising the promotion of locals into specialist roles, the Maldives can ensure that the economic benefits of its natural wealth are not only generated but also equitably retained within its communities [98,100].”

- Hanifaru seems to have much higher ray density. Why is that? Are protections working? If so, could those be applied to other areas to increase ray habitat and populations there and perhaps distribute visitor pressure across more viewing locations? Your numbers speak to implementing the same rules that Hanifaru has to other areas. Are any of the visitation rates limited by regulation, managing congestion and negative impact on ray health?

**Response:** Changed. We have updated the *Management and sanctuaries* section in the *Discussion* to clarify that while Hanifaru’s manta ray density is driven by unique conditions, its management success provides a vital proof-of-concept for the rest of the Maldives.

*Discussion*:

“Management and sanctuaries

There has never been a targeted commercial fishery for manta rays in the Maldives; however, these populations are still vulnerable to bycatch, illegal fishing, habitat degradation, and unregulated tourism [36,69,70]. The Maldivian government’s recognition of the economic value of iconic animals such as manta rays, whale sharks, and reef sharks, was instrumental in developing legal frameworks and policies such as the Hanifaru MPA, and the protection of shark and ray species [35,36]. Importantly, successful conservation relies on community support; a lack of buy-in and top-down conservation approaches can lead to non-compliance or policy reversal [105]. Recent attempts to reestablish longline fisheries and legalise shark fishing in the Maldives in 2024 threatened the sanctuary status, but were successfully blocked through community pushback and an online poll [106,107]. Yet in 2025, the government reopened the gulper shark (*Centrophorus* spp.) fishery, an action met with complex attitudes and a strong community opposition [108]. These events underscore the need for robust conservation strategies that align with national conservation goals with international commitments to the United Nations Convention on Biological Diversity (CBD) and the Conservation of Migratory Species of Wild Animals (CMS).

Nationwide there are 42 MPAs in the Maldives covering 116.3 km^2^ (0.5% of the 21,596 km^2^ of Economic Exclusive Zone), yet for many years Hanifaru remained the only MPA with active, site-specific monitoring and enforcement [36,58,69]. The high density and predictability of manta rays at Hanifaru MPA (mean of 32 individuals per trip) is driven by the geomorphological and oceanographic conditions, eddies that trap high concentrations of their zooplankton prey, making it a globally unique aggregation [109]. Although such densities of manta rays infrequently occur elsewhere in the Maldives, the Hanifaru MPA model demonstrates how rigorous regulation can successfully mitigate the impacts of high visitor pressure [69]. Since its designation in 2009, regulations have limited congestion by capping the number of visitors at 80 snorkellers and five boats at any one time, prohibiting SCUBA diving, enforcing a code-of-conduct and requiring licensed guides [69,110]. In 2012, the entirety of Baa Atoll was formally designated as the Baa Atoll Biosphere Reserve, with Hanifaru MPA as a core protected zone. Revenue generated through the Biosphere Reserve supports the Baa Atoll Conservation Fund, which is utilised for community education and research [110].

In 2025, drawing from the success of Hanifaru MPA, long-term monitoring and stakeholder consultation led to the introduction of active monitoring and enforcement of the South Ari Marine Protected Area (SAMPA), a critical aggregation area for whale sharks [111-113]. By implementing regulations and revenue-generating mechanisms modelled after Hanifaru MPA at other key megafauna aggregation sites, the government could prevent the degradation of high-traffic habitats while maintaining high-quality tourism experiences. A significant step towards this national scaling occurred in 2023 with the development of the Protected Species Regulation (2021/R-25), which provides a mandatory code of conduct for snorkellers, divers and boats involved in activities with protected species nationwide, including MRW, regardless of the MPA status [114]. This tiered approach, combining broad national conduct standards with intensive, site-specific management at high-value aggregation hubs, offers a pragmatic pathway to safeguard the biological health of the species while securing the long-term economic prosperity of the MRW industry.”

Reviewer #2: All my comments have been satisfactorily addressed by the authors.
Publications on manta ray tourism values in the Maldives are mostly non-species-specific, and some targeting manta rays are outdated, making this publication an original and updated source of information.
The authors acquired and stated the ethics permit and permit exemption required and ensured the anonymity of respondents in the MS.

**Response:** Thank you for your positive and helpful feedback.

7. PLOS authors have the option to publish the peer review history of their article ([what does this mean?](https://journals.plos.org/plosone/s/editorial-and-peer-review-process#loc-peer-review-history)). If published, this will include your full peer review and any attached files.

If you choose “no”, your identity will remain anonymous but your review may still be made public.


**Do you want your identity to be public for this peer review?** For information about this choice, including consent withdrawal, please see our [Privacy Policy](https://www.plos.org/privacy-policy).

Reviewer #1: No

Reviewer #2: No
